# Supplementary material for: Multiplexed single-cell transcriptomics reveals diverse phenotypic outcomes for pathogenic SHP2 variants
Source: Sci Adv. 2026 May 22;12(21):eaea9389. doi: 10.1126/sciadv.aea9389 (PMC13196782; doi:10.1126/sciadv.aea9389)
Supplement: Supplementary file 1 — Figs. S1 to S6 Legends for tables S1 to S3 [file sciadv.aea9389_sm.pdf]

Science Advances
AAAS

Supplementary Materials for

Multiplexed single-cell transcriptomics reveals diverse phenotypic outcomes for pathogenic SHP2 variants

Anne E. van Vlimmeren *et al.*

Corresponding author: José L. McFaline-Figueroa, jm5200@columbia.edu; Neel H. Shah, neel.shah@columbia.edu

Sci. Adv. **12**, eaea9389 (2026)
DOI: 10.1126/sciadv.aea9389

The PDF file includes:

Figs. S1 to S6
Legends for tables S1 to S3

Other Supplementary Material for this manuscript includes the following:

Tables S1 to S3

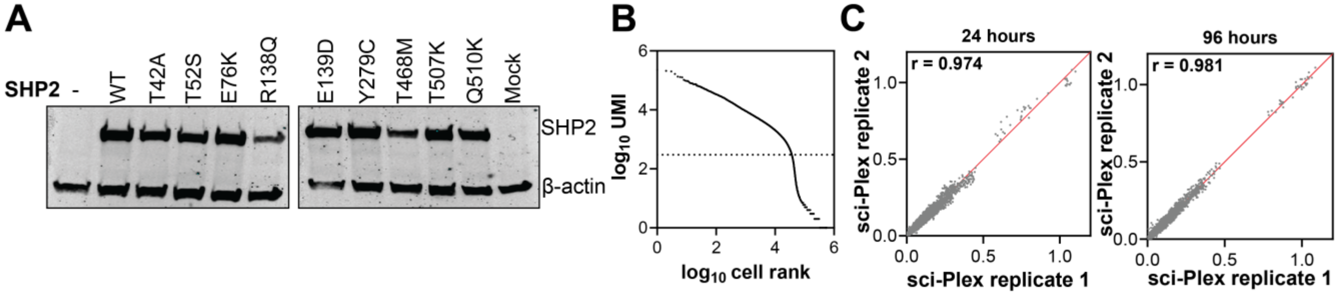

D sci-Plex SHP2 variant screen #1

Timepoint	Total Cell Count	Mean Cell Count per SHP2mut-EGF	Max Cell Count per SHP2mut-EGF	Min Cell Count per SHP2mut-EGF	Median UMI	Median Hash UMI
24hr	13,327	138.8	262	60	1054	52
96hr	16,034	167.0	404	21	1002	63
Total	29,361	152.9	404	21	1022	58

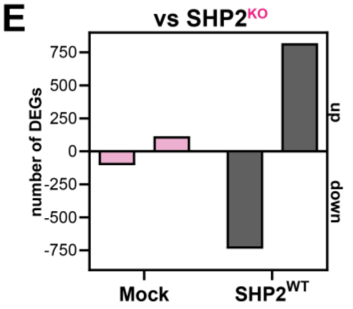

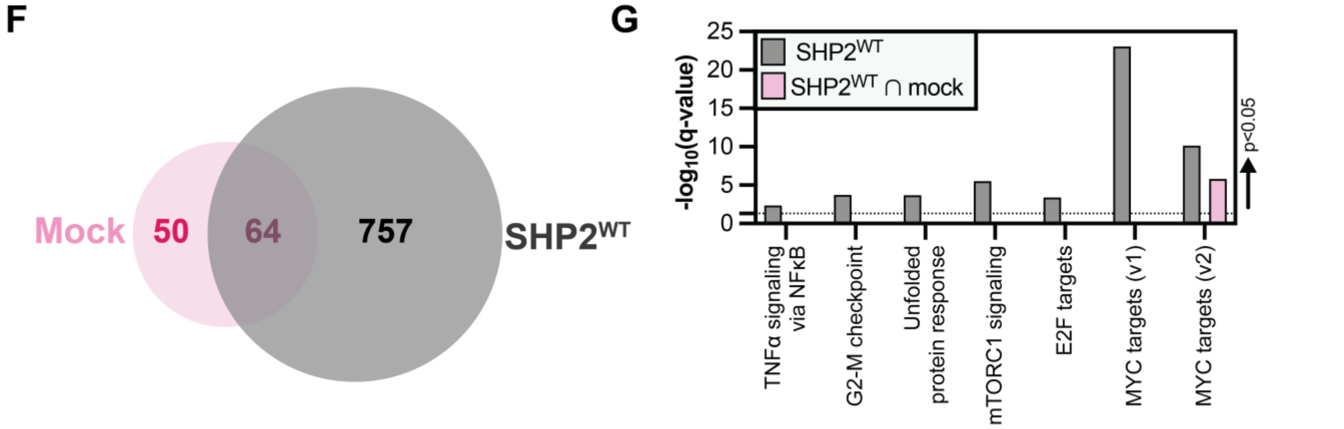

Figure S1. Comprehensive screen of pathogenic *PTPN11* mutations using sci-Plex. (**A**) Western blot showing expression of SHP2^{WT} and mutants transfected into SHP2^{KO} HEK293 cells. (**B**) Knee plot of unique molecular identifier (UMI) vs cell rank with a UMI cut-off of 300 to filter for high-quality cells (**C**) Correlation of the mean expression between two independent replicates at 24 hour (*left*) and 96 hours (*right*). Each point represents the mean expression of a single gene for a specific SHP2 variant, e.g. EGFR expression in SHP2^{R138Q} cells. (**D**) Table of experimental summary metrics for the preliminary screen after quality filters. (**E**) Number of differentially expressed genes (normalized effect size <-0.25 or >0.25, false discovery rate <0.05) for mock-transfected cells and SHP2^{WT}-expressing cells respectively, compared to SHP2^{KO} cells. Number of differentially expressed genes for SHP2 variants vs SHP2^{KO} can be found in **Figure 3E**. (**F**) Venn diagram of SHP2^{WT} and mock-transfected cells to visualize the intersection of upregulated DEGs compared to SHP2^{KO}. 64 detected DEGs are shared between the conditions. (**G**) Hypergeometric gene set enrichment analysis of unique SHP2^{WT} and shared SHP2^{WT}/mock-transfected DEG intersections. SHP2^{WT} upregulated DEGs are significantly enriched for cell cycle, mTOR, and MYC signaling.

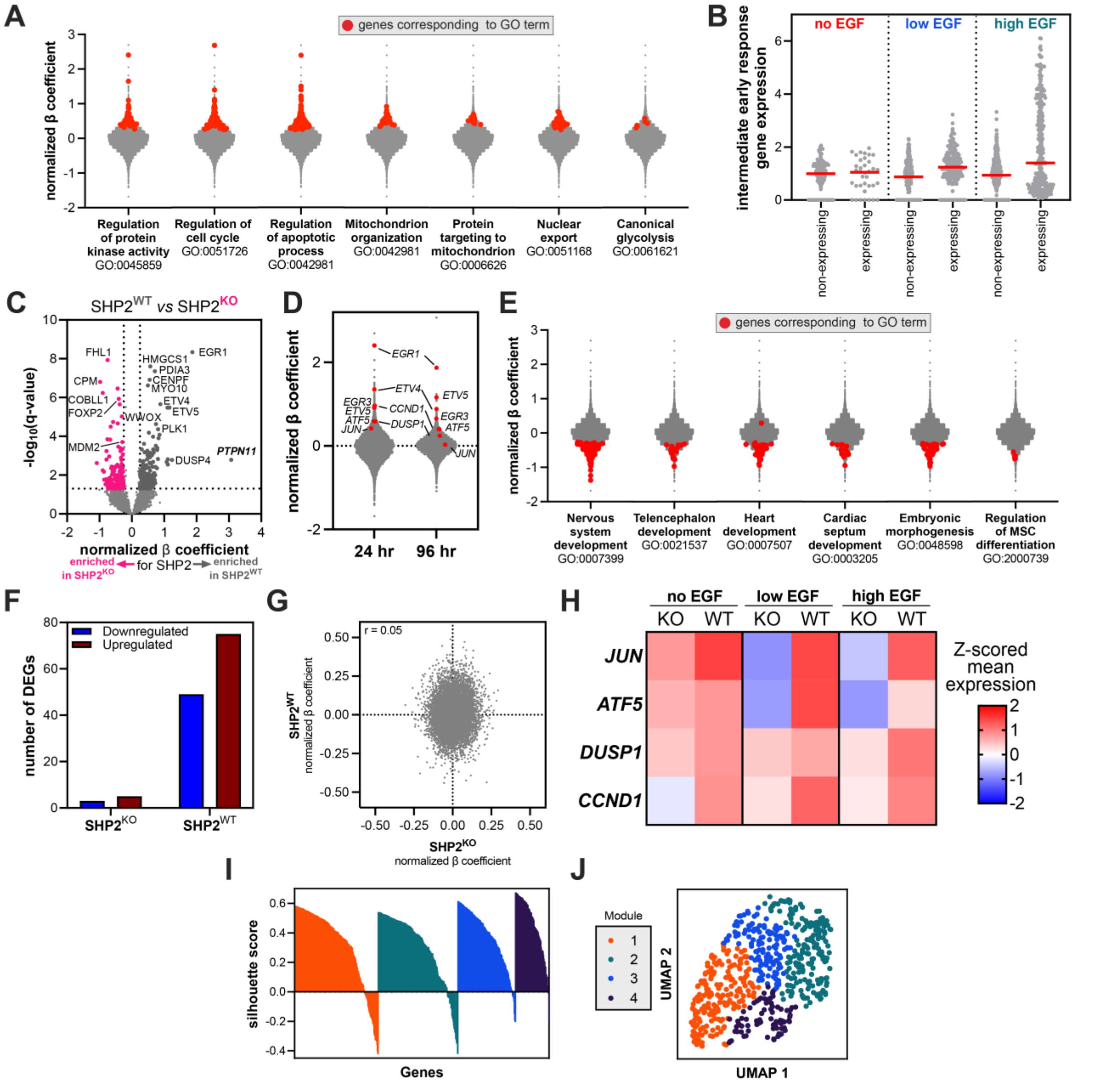

Figure S2. Comparison of SHP2^{WT} transcriptome to SHP2^{KO}. (**A**) Normalized effect sizes for differentially expressed genes (SHP2^{WT} vs SHP2^{KO}; false discovery rate < 0.05, normalized effect size > 0.25; red dots) corresponding to statistically enriched Gene Ontology (GO) terms. Genes related to transcription, translation, and RNA more broadly were removed prior to analysis. (**B**) Expression of immediate early response genes of non-SHP2 expressing cells in the SHP2^{WT} sample versus SHP2^{WT}-expressing cells. (**C**) Volcano plots showing SHP2-induced differentially expressed genes for SHP2^{KO} and SHP2^{WT} at 96 hours. Any significant transcript with a normalized effect size of > 0.25 or <-0.25 is colored. (**D**) Comparison of early response genes (*EGR1/3, ETV4/5, DUSP1, CCND1, ATF5, JUN*; red dots) at 24 hours and 96 hours between SHP2^{WT} (top of violin) and SHP2^{KO} (bottom of violin). (**E**) Same as (**A**), but for differentially expressed genes with false discovery rate < 0.05, normalized effect size < -0.25. MSC = mesenchymal stem cells. (**F**) Number of up- and down-regulated genes in response to EGF stimulation for SHP2^{KO} and SHP2^{WT}. The number of EGF-induced DEGs is larger for SHP2^{WT}. (**G**) Correlation of β coefficients for differentially expressed genes between SHP2^{KO} and SHP2^{WT}. Low β coefficient shows SHP2^{KO} and SHP2^{WT} are not correlated or inversely correlated but rather have their own gene expression effects. (**H**) Heatmap showing expression of early response genes with high basal expression in SHP2^{WT} unstimulated cells compared with SHP2^{KO} cells. (**I**) Silhouette plot of gene modules suggests that 4 models achieve good consistency within clusters and separation between clusters for most genes. (**J**) UMAP of gene space with 4 gene modules indicated

by color.

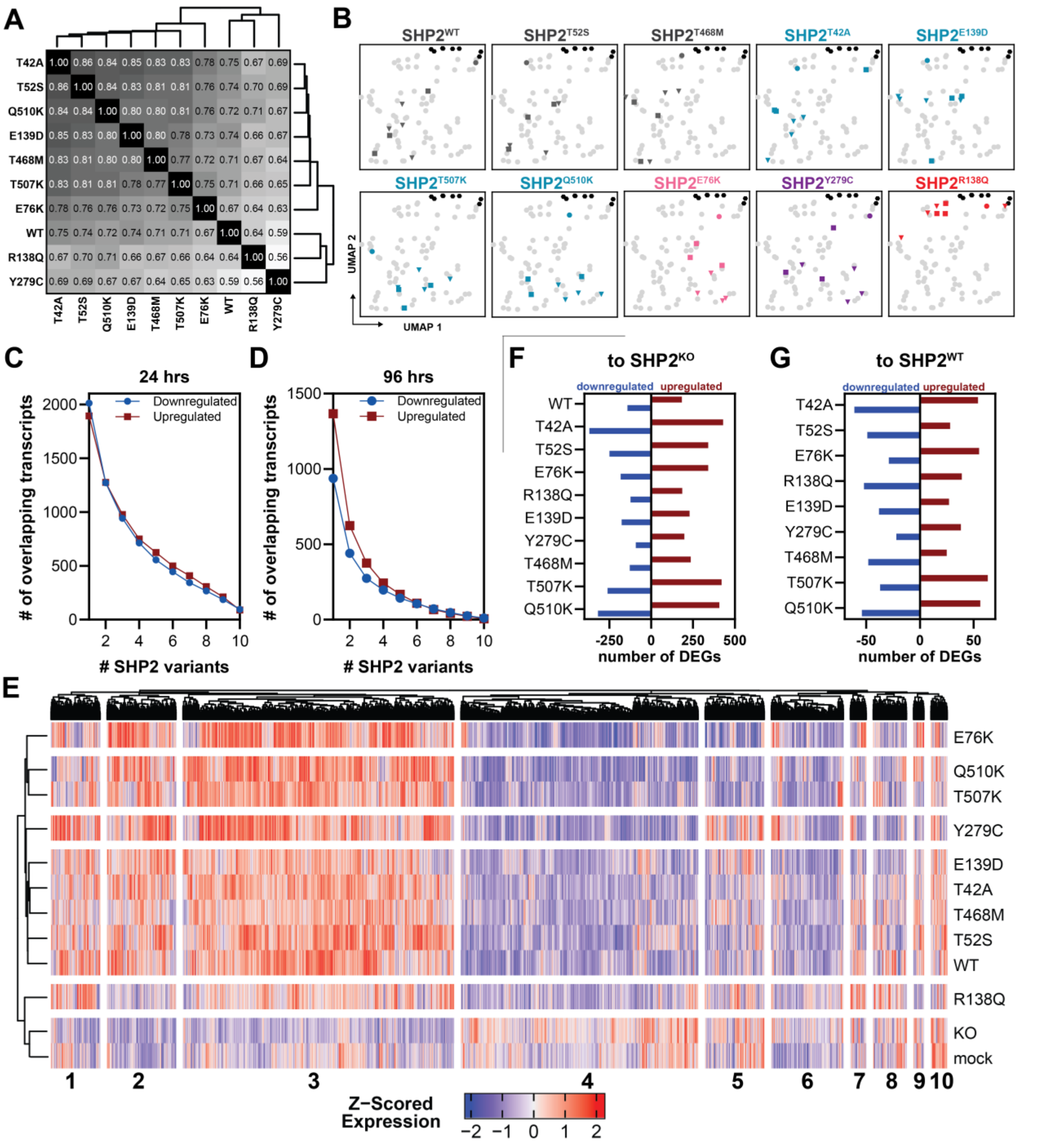

Figure S3. Analysis of shared and distinct SHP2 variant transcriptomes. (**A**) Heatmap of β coefficient correlation (pearson's ρ) with unsupervised hierarchical clustering, comparing SHP2^{WT} and all SHP2 variants at 96 hours. SHP2^{Y279C}-expressing cells appear most distinct. (**B**) Pseudo-bulked $\log_2$ fold-change expression of cells grouped by 24 hour time point, SHP2 variant and EGF dose against unstimulated SHP2^{KO} cells. Black hexagons represent mock-transfected cells. Circles indicate no EGF, squares indicate low EGF concentration and triangles indicate high EGF concentration. (**C**) Number of overlapping genes that are significantly enriched (normalized effect size <-0.25 or >0.25, and false discovery rate < 0.05) over the SHP2^{KO} control at 24 hours. (**D**) Same as (**C**), but for 96 hours. (**E**) Heatmap of z-scored pseudo-bulked expression of 24 hour high EGF-stimulated cells for the union of DEGs. Clustering of mutants demonstrates the shared differences of SHP2 mutants compared to SHP2^{KO} and mock-transfected groups. SHP2^{R138Q} clustered distinctly from the other SHP2 variants. Gene modules are numbered and genes for each module can be found in **Table S3**. (**F**) Number of differentially expressed genes (normalized effect size <-0.25 or >0.25, and false discovery rate < 0.05) per SHP2 mutant, compared to SHP2^{KO},

at 96 hours after EGF stimulation. (**G**) Same as (**F**), but for each SHP2 variant against SHP2^{WT}.

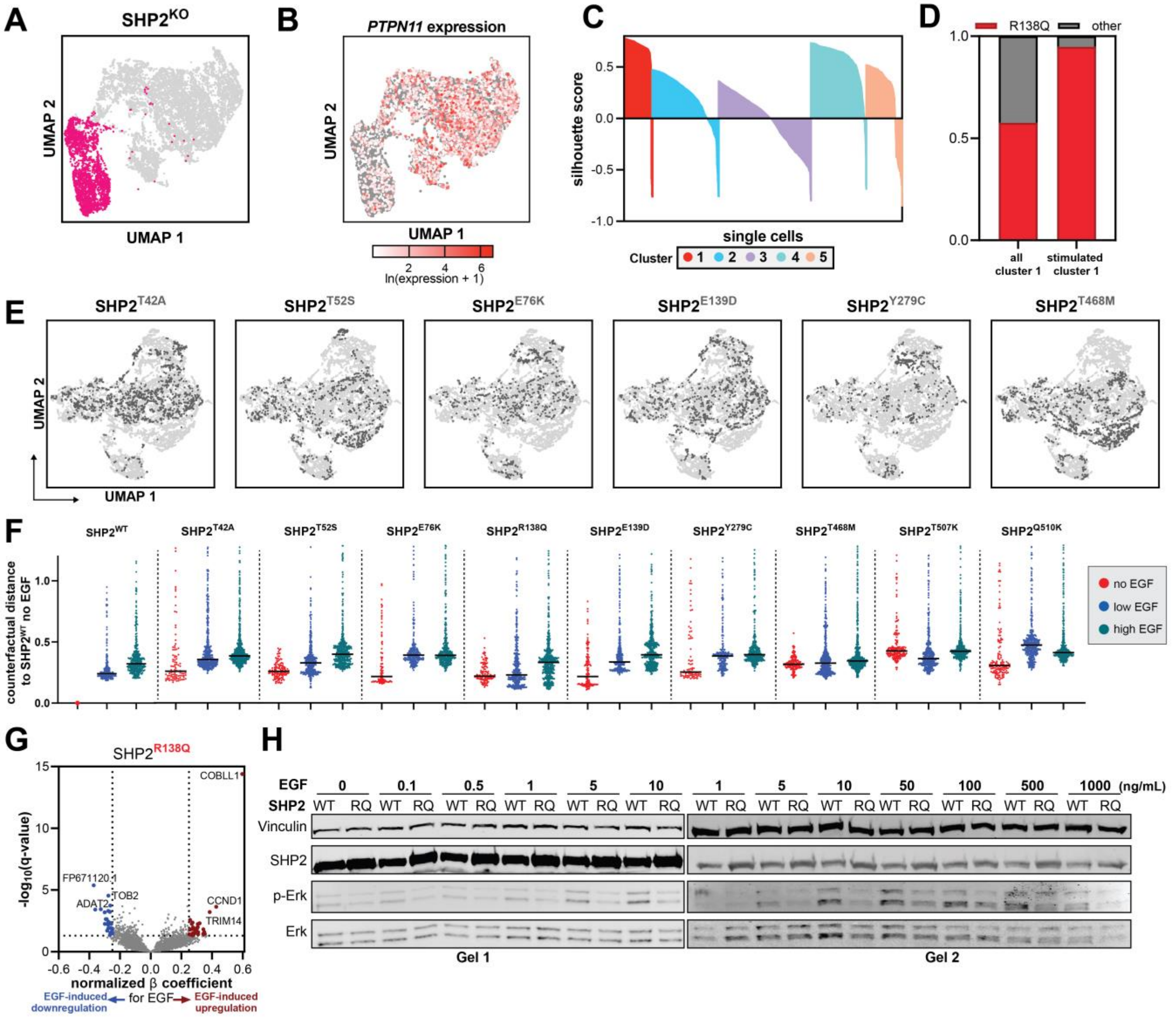

Figure S4. **MrVI analysis of SHP2 variant response to EGF stimulation.** (**A**) UMAP of the MrVI Z-space for single cells shows distinct grouping for SHP2^{KO} (*pink*) compared to any SHP2 variant (*light grey*). (**B**) UMAP of the latent sample-aware z-space of 24 hour post-EGF SHP2 variant cells. log(*PTPN11*) expression is indicated on a white-red color gradient, with the non-expressing cells colored in light grey. Variation in *PTPN11* expression is largely uniform across the space. (**C**) Silhouette plot of Leiden-based clustering in cell space suggests that 5 clusters achieve good consistency within clusters and separation between clusters for most cells. (**D**) Mutant per cluster distribution for cluster 5 demonstrates disproportionate representation of SHP2^{R138Q}. (**E**) UMAPs of MrVI Z-space for cells expressing different SHP2 variants (*dark grey*). (**F**) Distribution of MrVI-derived counterfactual distance between each mutant and unstimulated SHP2^{WT} cells shows distinct behaviors in response to EGF dose: positively correlated (SHP2^{WT}, SHP2^{T42A}, SHP2^{T52S}, SHP2^{E139D}), early saturating (SHP2^{E76K}, SHP2^{Y279C}), desensitized (SHP2^{R138Q}), unaffected (SHP2^{T468M}), and irregular (SHP2^{T507K}, SHP2^{Q510K}). (**G**) Volcano plot showing EGF-induced differentially expressed genes for SHP2^{R138Q}. Any significant (false discovery rate < 0.05) transcript with a normalized effect size (NES) of > 0.25 or <-0.25 is colored. (**H**) Representative western blot of dose-response curves for SHP2^{WT} and SHP2^{R138Q} (n = 3 independent transfections).

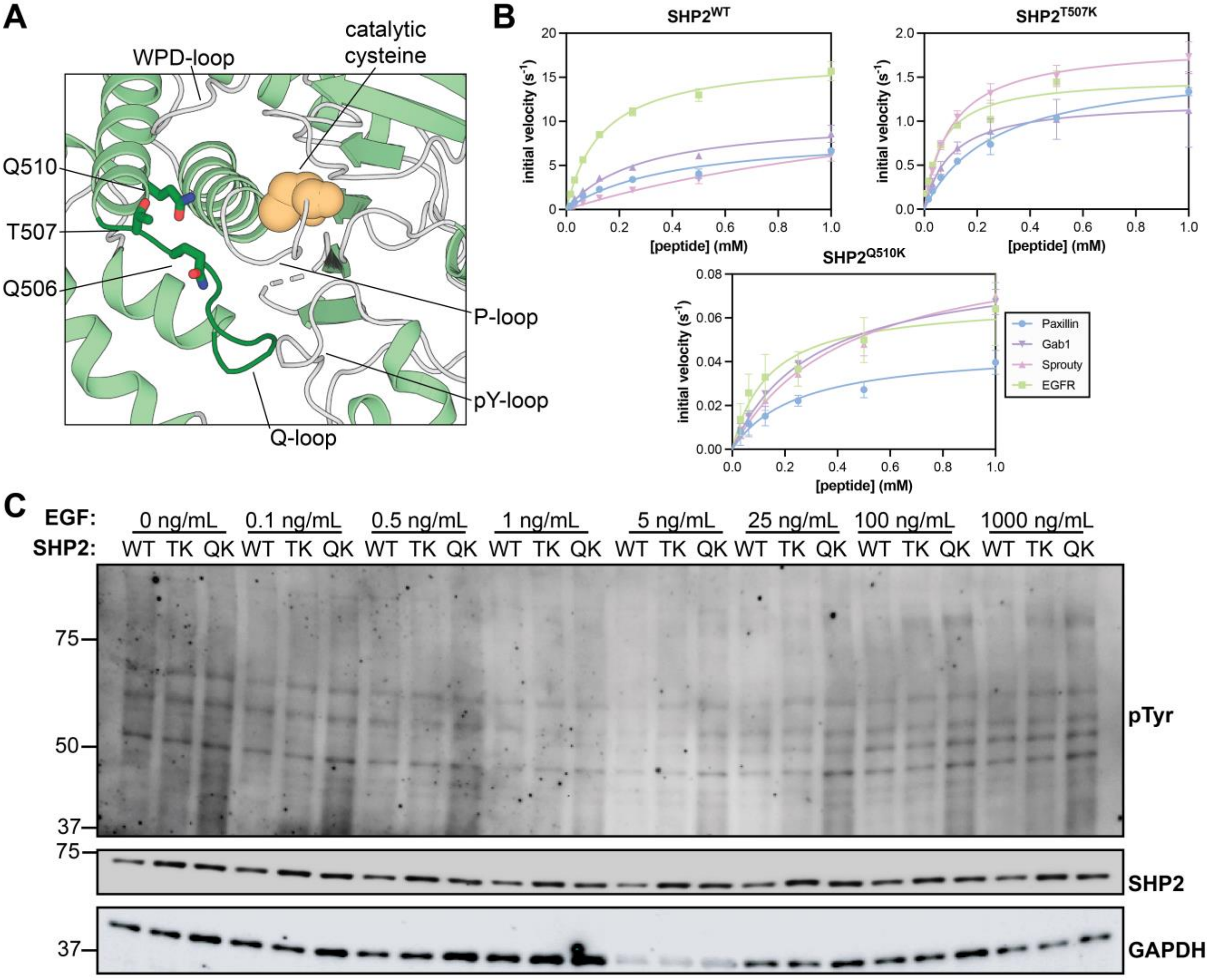

Figure S5. Biochemical and cellular characterization of SHP2^{T507K} and SHP2^{Q510K}. (**A**) Structure of SHP2 catalytic pocket. Catalytic cysteine and key catalytic loops are indicated (pY-loop: residues 276-282, WPD-loop: residues 420-429, P-loop: residues 458-465, and Q-loop: residues 501-507). Q510, T507, and Q506 are indicated as sticks. (**B**) Michaelis-Menten curves for PTP^{WT}, PTP^{T507K} and PTP^{Q510K} with four different peptides. Data are derived from averages of 3 or more independent peptide and protein dilutions and measurements. (**C**) Representative western blot of global phosphotyrosine levels as a function of EGF dose for cells expressing SHP^{WT} (WT), SHP2^{T507K} (TK), and SHP2^{Q510K} (QK) (n = 3 independent transfections). SHP2^{Q510K} shows increased phosphorylation of tyrosine at every EGF dose.

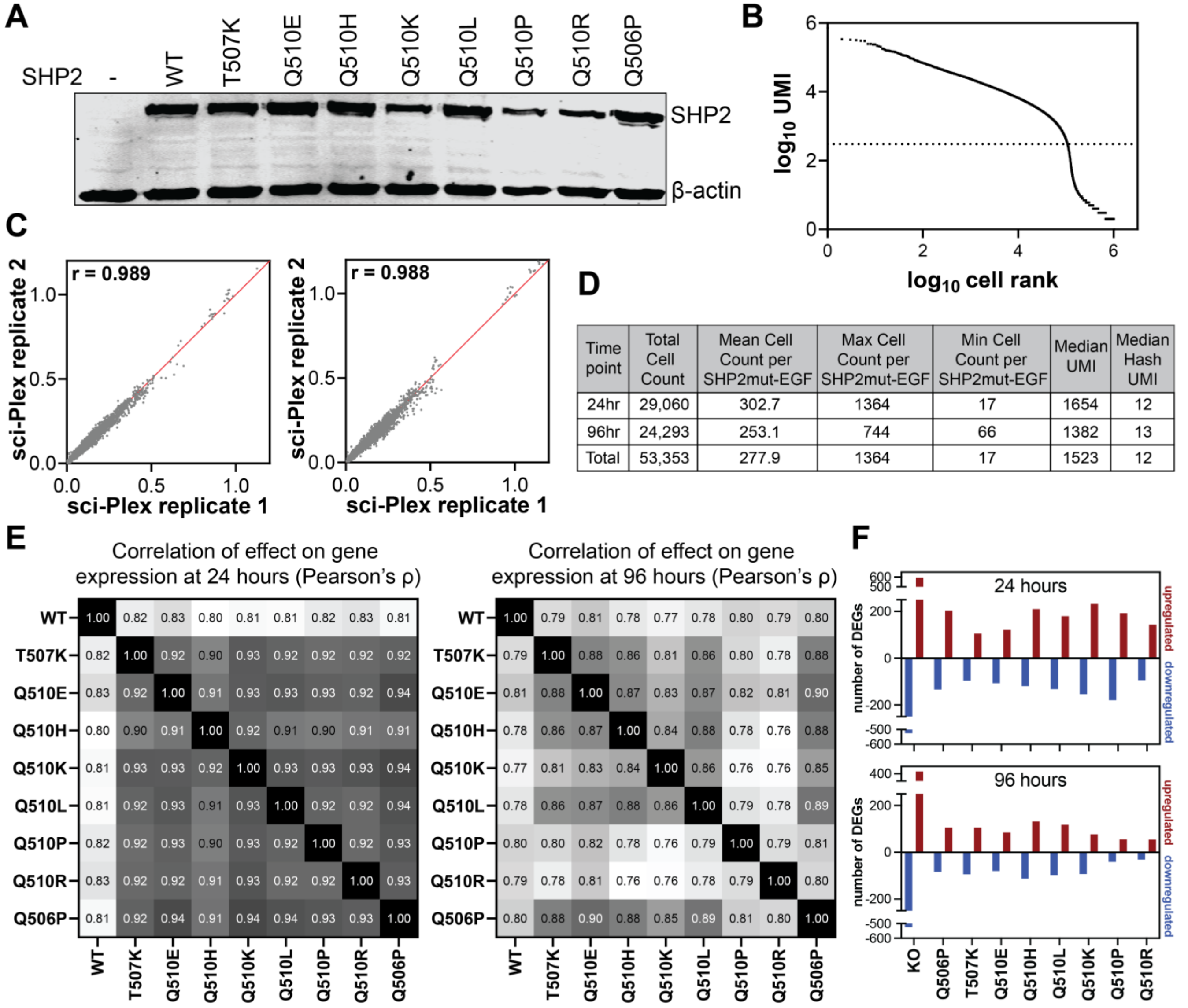

Time point	Total Cell Count	Mean Cell Count per SHP2mut-EGF	Max Cell Count per SHP2mut-EGF	Min Cell Count per SHP2mut-EGF	Median UMI	Median Hash UMI
24hr	29,060	302.7	1364	17	1654	12
96hr	24,293	253.1	744	66	1382	13
Total	53,353	277.9	1364	17	1523	12

Figure S6. Screening of Q-loop mutants. (A) Western blot shows expression of SHP2^{WT} and mutants included in the Q510 follow-up screen, compared to parental SHP2^{KO} cell line. (**B**) Knee plot as UMI vs cell rank with a UMI cut-off of 300. (**C**) Correlation (pearson's ρ) between two independent replicates at 24 hours (*left*) and 96 hours (*right*). (**D**) Table of experimental summary metrics. (**E**) Correlation of β coefficients of SHP2 variants in Q510 screen at 24 hours (*left*) and 96 hours (*right*). (**F**) Number of differentially expressed genes (normalized effect size <-0.25 or >0.25, false discovery rate <0.05) for each SHP2 variant against SHP2^{WT} at 24 hours (*top*) and 96 hours (bottom).

Supplementary Tables (included as separate spreadsheet files)

Table S1. SHP2-driven effects in gene expression. (a) Differentially expressed genes test (SHP2 coefficients) for $SHP2^{WT}$ vs $SHP2^{KO}$. (b) Gene Ontology analysis of DEGs upregulated in $SHP2^{WT}$. (c) Gene Ontology analysis of DEGs downregulated in $SHP2^{WT}$. (d) GSEA analysis of $SHP2^{WT}$ vs $SHP2^{KO}$.

Table S2. EGF-driven effects in gene expression. (a) EGF-induced differentially expressed genes for each sample. (b) Gene Modules.

Table S3. Gene expression effects driven by SHP2 mutants. (a) Structural, biochemical, and clinical effects of SHP2 variants. (b) Differentially expressed gene test for all SHP2 variants vs $SHP2^{KO}$. (c) Gene modules based on clustering of SHP2 variant DEGs compared to KO. (d) Differentially expressed gene test for all SHP2 mutants vs $SHP2^{WT}$. (e) Common transcriptome genes separated by time point and differential direction. (f) Differentially expressed genes test for $SHP2^{Q510K/R}$ vs $SHP2^{WT}$.
