# Supplementary material for: Multiplexed single-cell transcriptomics reveals diverse phenotypic outcomes for pathogenic SHP2 variants
Source: Sci Adv. 2026 May 22;12(21):eaea9389. doi: 10.1126/sciadv.aea9389 (PMC13196782; doi:10.1126/sciadv.aea9389)
Supplement: Supplementary file 2 [file sciadv.aea9389.v1.pdf]

MOLECULAR BIOLOGY

Multiplexed single-cell transcriptomics reveals diverse phenotypic outcomes for pathogenic SHP2 variants

Anne E. van Vlimmeren[1,2]†, Ross M. Giglio[3,4]†, Ziyuan Jiang[1], Minhee Lee[1], José L. McFaline-Figueroa[4,5,6]*, Neel H. Shah[1,6]*

Copyright © 2026 The Authors, some rights reserved; exclusive licensee American Association for the Advancement of Science. No claim to original U.S. Government Works. Distributed under a Creative Commons Attribution License 4.0 (CC BY).

The protein tyrosine phosphatase SHP2, encoded by *PTPN11*, is an important regulator of Ras/mitogen-activated protein kinase signaling that acts downstream of receptor tyrosine kinases and other transmembrane receptors. Germline *PTPN11* mutations cause developmental disorders such as Noonan syndrome, whereas somatic mutations drive various cancers. While many pathogenic mutations enhance SHP2 catalytic activity, others are inactivating or affect protein interactions, confounding our understanding of SHP2-driven disease. Here, we combine single-cell transcriptional profiling of cells expressing clinically diverse SHP2 variants with protein biochemistry, structural analysis, and cell biology to explain how pathogenic mutations dysregulate signaling. Our analyses reveal that loss of catalytic activity does not phenocopy SHP2 knockout at the gene expression level, that some mechanistically distinct mutations have convergent phenotypic effects, and that different mutations at the same hotspot residue can yield divergent cell states. These findings provide a framework for understanding the connection between SHP2 structural perturbations, cellular outcomes, and human diseases.

INTRODUCTION

SHP2, encoded by *PTPN11*, is a ubiquitously expressed protein tyrosine phosphatase (PTP) in humans that functions as a signaling hub downstream of many transmembrane receptors and has critical roles in cell proliferation, cell differentiation, immunity, and development (Fig. 1A). *PTPN11* missense mutations drive many human diseases, including hematopoietic malignancies such as acute myeloid leukemia (AML), acute lymphoid leukemia (ALL), and juvenile myelomonocytic leukemia (JMML) (Fig. 1B) (*1–3*). Association with solid tumors such as neuroblastoma, hepatocellular carcinoma, glioblastoma, and melanoma has also been described (*4–6*). Germline mutations in *PTPN11* underlie congenital disorders, including approximately 50% of cases of Noonan syndrome (NS) (*7–11*) and 95% of NS with multiple lentigines (NSML) cases (Fig. 1B) (*12–14*). NS is characterized by facial dysmorphia, intellectual disability, and heart defects—in particular, pulmonic stenosis (*7*). In addition to these NS phenotypes, patients with NSML have a high incidence of hypertrophic cardiomyopathy, electrocardiographic abnormalities, and hearing loss (*14*).

Most disease-associated functions of SHP2 have been attributed to its role in Ras/mitogen-activated protein kinase (MAPK) signaling. Both NS and NSML are categorized as "RASopathies" and share similarities with other syndromes caused by mutations in Ras/MAPK components. In many cancers, SHP2 mediates signal transduction from receptor tyrosine kinases to Ras, and several allosteric inhibitors of SHP2 have entered clinical trials for the treatment of receptor tyrosine kinase–driven cancers (*15*, *16*). SHP2 promotes Ras/MAPK signaling through several mechanisms, including inhibition of Sprouty1, a negative regulator of Ras, direct dephosphorylation and activation of Ras, and dephosphorylation of scaffold proteins to prevent the recruitment of Ras guanosine triphosphatase (GTPase)–activating proteins to signaling complexes (*17–20*). SHP2 functions downstream of a variety of transmembrane receptors and can activate not just the Ras/MAPK pathway but also phosphatidylinositol 3-kinase signaling, Janus kinase/signal transducers and activators of transcription signaling, and immune checkpoint signaling (Fig. 1A) (*21–24*).

Hundreds of SHP2 mutations are cataloged in clinical databases (Fig. 1C), and these mutations disrupt SHP2 structure and function through diverse mechanisms (*25–29*). SHP2 is canonically activated by binding to phosphoproteins, which disrupts its resting autoinhibited state to yield an active enzyme (Fig. 1A) (*30*, *31*). Many oncogenic mutations also disrupt autoinhibition, leading to catalytic gain-of-function effects (*25*, *31*). By contrast, other mutations appear to act through noncatalytic mechanisms (*32*). Despite extensive studies, how these molecular effects translate into disease phenotypes remains unclear. One emerging theme is that SHP2 mutations can alter protein-protein interactions, as seen in NSML-associated variants. Many NSML mutations result in low or no catalytic activity but also cause large conformational changes that enhance binding to *MPZL1*/Pzr, a driver of hypertrophic cardiomyopathy through the Akt and nuclear factor κB pathways (*33*). Our recent work suggests that many pathogenic mutations in SHP2 broadly reshape its protein interaction network, yet the transcriptional and signaling consequences of these changes remain poorly understood (*27*, *34*).

Subtle perturbations to the structure of a signaling protein, such as those caused by missense mutations, can propagate to changes in protein-protein interactions and proximal signaling events, which in turn, can alter downstream gene expression. A previous study on the transcriptomes of cells expressing two SHP2 mutants that disrupt autoinhibition found increased expression of metabolic proteins, highlighting the potential insights that could be gained from studying mutation-specific transcriptional changes (*35*). In addition, profiling mutation-driven changes in gene expression may also uncover key disruptions to protein function and can be leveraged to aid our understanding of SHP2 structure-function relationships.

[1]Department of Chemistry, Columbia University, New York, NY 10027, USA. [2]Department of Biological Sciences, Columbia University, New York, NY 10027, USA. [3]Department of Molecular Pharmacology and Therapeutics, Columbia University Medical Center, New York, NY 10032, USA. [4]Department of Biomedical Engineering, Columbia University, New York, NY 10027, USA. [5]Irving Institute for Cancer Dynamics, Columbia University, New York, NY 10027, USA. [6]Herbert Irving Comprehensive Cancer Center, Columbia University, New York, NY 10032, USA.
*Corresponding author. Email: jm5200@columbia.edu (J.L.M.-F.); neel.shah@columbia.edu (N.H.S.)
†These authors contributed equally to this work.

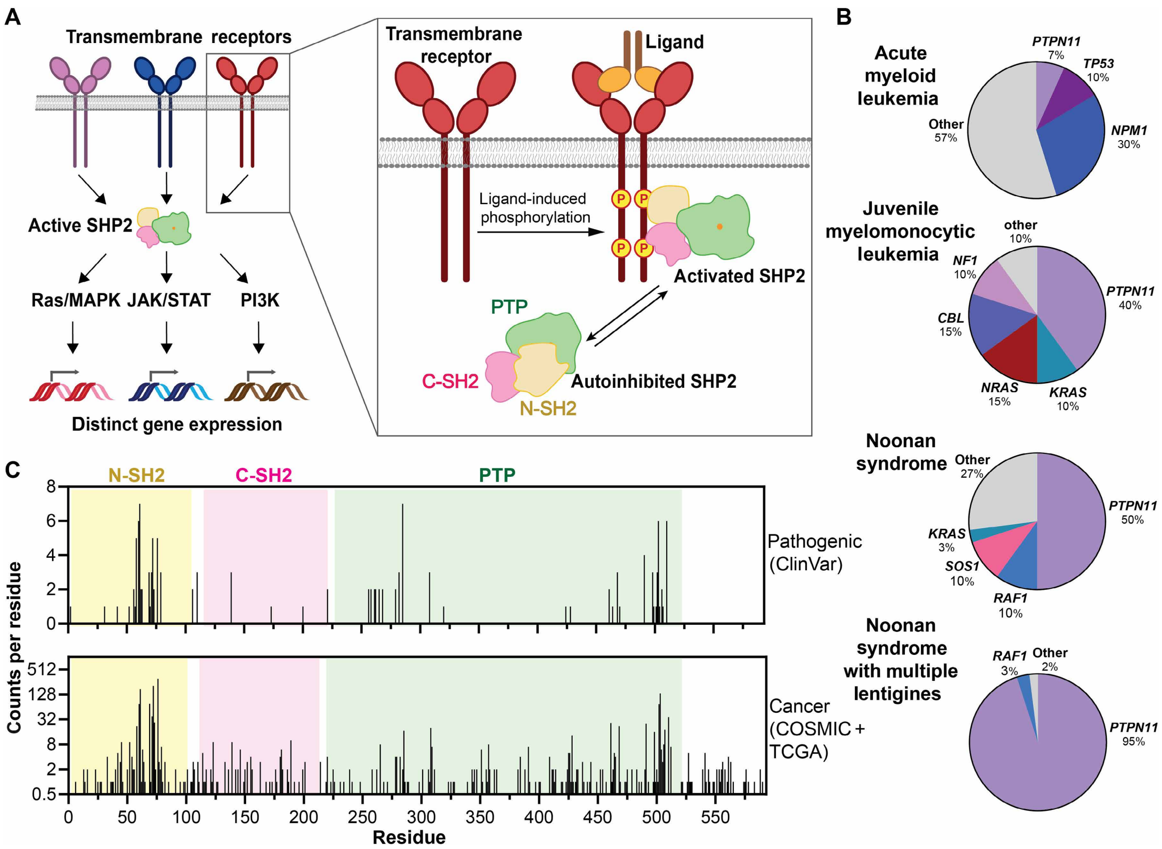

Fig. 1. Biological function and pathology of SHP2. (**A**) SHP2 receives input from a variety of cell signaling pathways, and SHP2 activation by binding to phosphoproteins has a diverse array of potential signaling and transcriptional outcomes. (**B**) Pie charts showing disease-driving genes for various human diseases, as identified in DNA sequencing of patient cohorts (*2*, *7–12*). *PTPN11* mutations underlie both congenital disorders and cancers. (**C**) Positions and frequencies of missense mutations in SHP2 along its 593-residue sequence. Pathogenic mutations were obtained from the ClinVar dataset. Cancer-associated mutations were obtained from the COSMIC and TCGA databases.

Multiplexed single-gene mutation studies have yielded insights into variants in genes like *TP53* (*36*), *KRAS* (*37*), *PAX5* (*38*), *RUNX1* (*39*), and *GATA1* (*40*). Here, we use single-nucleus RNA sequencing to map the transcriptional impact of 15 clinically and mechanistically diverse pathogenic *PTPN11* mutations across two independent screens. We identify SHP2 presence as a critical driver for the cellular response to epidermal growth factor (EGF) stimulation and demonstrate that the Arg138→Gln (R138Q) mutation, which prevents C-SH2 domain interactions, attenuates EGF-driven signaling independent of catalytic activity. Further, we show that two mutations, Thr507→Lys (T507K) and Gln510→Lys (Q510K), have convergent effects on the biochemical and transcriptional level, with charge of the resulting amino acid as a likely driver. Last, we show that different disease-relevant substitutions at catalytic residue Q510 have different effects on SHP2 structure and activity, propagating to distinct transcriptional outcomes. By systematically profiling the transcriptional landscape of *PTPN11* variants, we provide insights into how SHP2 mutations alter protein function.

RESULTS

Single-cell transcriptomics identifies global transcriptional changes induced by SHP2 expression

To profile the effects of a collection of nine pathogenic SHP2 mutations on gene expression networks alone and under mitogen stimulation, we used sci-Plex-v2 multiplex single-cell RNA (scRNA) sequencing (*41*, *42*). We transfected either $SHP2^{WT}$ or mutant SHP2 into an SHP2 knockout ($SHP2^{KO}$) human embryonic kidney (HEK) 293 cell line (a total 12 distinct transfection conditions) (Fig. 2, A and B, and fig. S1A). Cells were stimulated with a range of EGF concentrations or left unstimulated (a total of eight different concentrations) for 24 or 96 hours (two time points), as we hypothesized on the basis of previous studies that we would identify differential sustained or feedback programs at these time points that would allow for clear identification of mutant differences (*43*). Nuclei for each condition were uniquely barcoded by fixation of an oligonucleotide hash. Barcoded nuclei were pooled, cDNA processed, and single-nuclei mRNA libraries were generated using our modified version of combinatorial indexing RNA sequencing (*41*, *42*, *44–47*). We captured a total of 29,716 cells across two replicates with a mean of 2447 cells per SHP2 variant and a mean coverage of 155 cells per unique combination of SHP2 variant, EGF dose, and time point (fig. S1, B to D).

First, we established the effect of $SHP2^{WT}$ presence on gene expression by comparing $SHP2^{KO}$ and $SHP2^{WT}$ cells across EGF-stimulation conditions (Fig. 2C and table S1). We juxtaposed this with a comparison between $SHP2^{KO}$ and mock-transfected cells as our control (Fig. 2D). Between mock-transfected cells and $SHP2^{KO}$ cells, we identified 114 genes as significantly up-regulated [quasi-Poisson regression, >0.25 β coefficient, <0.05 false discovery rate (FDR)] in mock-transfected cells, and 105 genes that were down-regulated

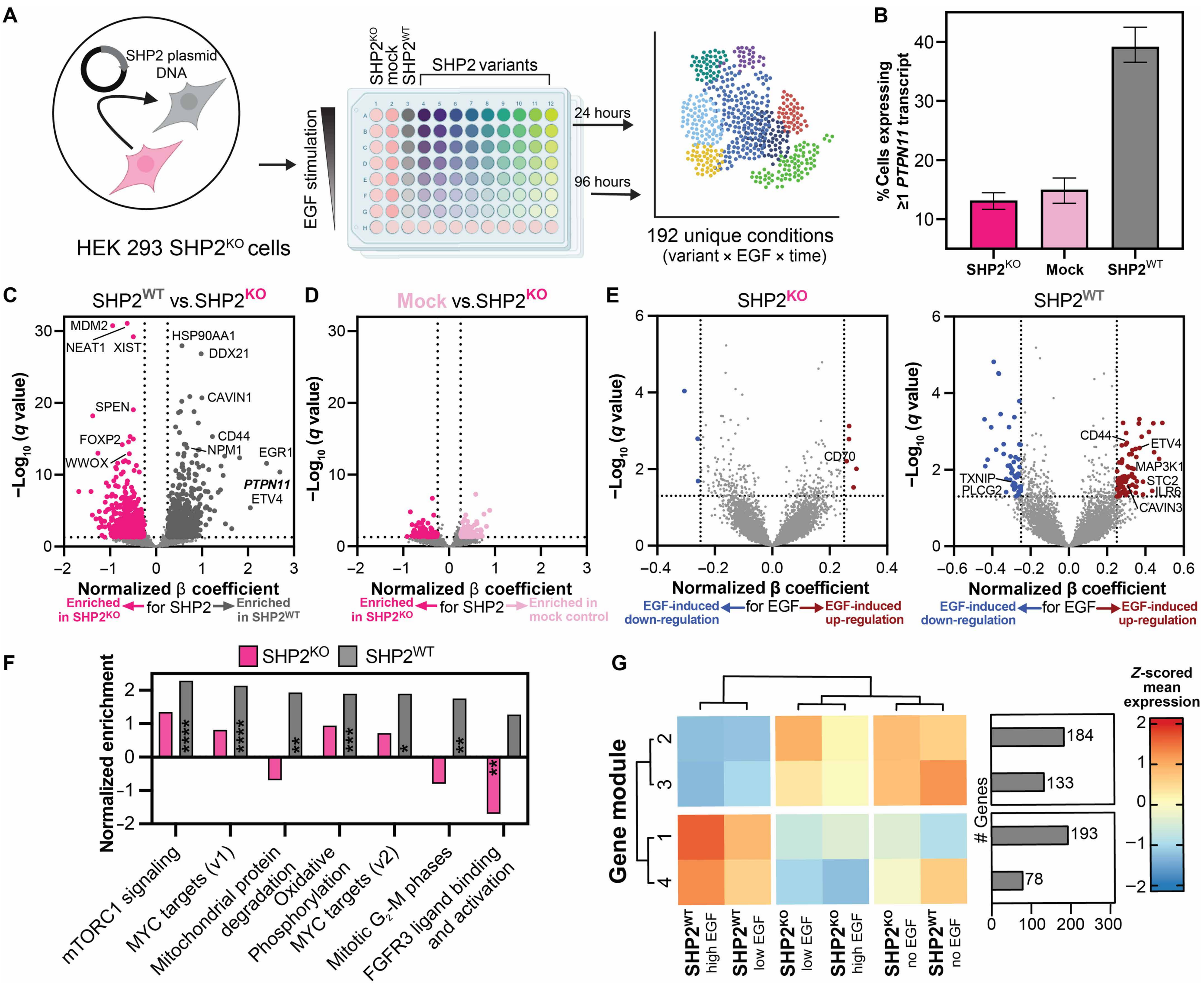

Fig. 2. Single-nucleus RNA sequencing reveals the transcriptional effect of SHP2 expression. (**A**) Schematic overview of RNA sequencing experiment to probe effects of SHP2 on gene expression. (**B**) Percentage of *PTPN11*-expressing cells in the SHP2^{KO} population, SHP2^{WT}-transfected cells, and mock-transfected cells, of the total number of cells sequenced for those respective samples. (**C**) Volcano plots showing SHP2-induced DEGs for SHP2^{KO} and SHP2^{WT} at 24 hours. Any significant (FDR < 0.05) transcript with a β coefficient of >0.25 or < −0.25 is colored. (**D**) The same as (C), but for mock-transfected versus SHP2^{KO}. (**E**) Volcano plots showing EGF-induced DEGs for SHP2^{KO} (top) and SHP2^{WT} (bottom). Any significant (FDR < 0.05) transcript with a normalized β coefficient of >0.25 or < −0.25 is colored. (**F**) GSEA shows pathways of DEGs for SHP2^{WT} and SHP2^{KO}. * denotes FDR < 0.05, ** < 0.01, *** < 0.001, and **** < 0.0001. (**G**) Four gene modules were identified between SHP2^{WT} and SHP2^{KO}. SHP2^{WT} without EGF stimulation behaves most similar to SHP2^{KO}. Low EGF is defined as 12.5 to 50 ng/ml; high EGF is defined as 100 to 1000 ng/ml.

(<−0.25 β coefficient, <0.05 FDR) (Fig. 2D and fig. S1E). By contrast, we identified 820 genes that were significantly up-regulated in cells expressing SHP2^{WT}, including *PTPN11* itself (Fig. 2C and fig. S1E). Of these 820 genes, only 64 overlap with up-regulated genes in the mock-transfected sample, indicating small levels of noise associated with transfection (fig. S1, F and G). Genes up-regulated for SHP2^{WT} were enriched for Gene Ontology terms related to kinase signaling and cell cycle, as well as nuclear export and mitochondrial import (fig. S2A and table S1). Moreover, several known EGF-response genes (*48*, *49*) were also enriched, including *EGR1/3*, *ETV4/5*, *JUN*, *ATF5*, *CCND1*, and *DUSP1*, indicating that the mere presence of SHP2 promotes the cellular response to EGF stimulation (Fig. 2C and fig. S2B). This is consistent with the observation that receptor tyrosine kinase–driven cancer cell lines depend on SHP2 for proliferation (*50*). These genes remained highly expressed in SHP2^{WT} cells compared to SHP2^{KO} at 96 hours (fig. S2, C and D). A total of 738 genes were down-regulated in SHP2^{WT}-expressing cells compared to SHP2^{KO} (Fig. 2C and table S1). Notably, we detected altered expression of genes related to heart development, mesenchymal stem cell (MSC) differentiation, and telencephalon development in SHP2^{KO} cells, aligning with the known roles of SHP2 in cardiac pathology, MSC regulation, and neurodevelopment (fig. S2E and table S1) (*51*–*53*). Collectively, these findings indicate that our approach detects diverse and disease-relevant SHP2-induced transcription in our model cell line.

SHP2^{WT} expression shapes the cellular response to EGF stimulation

Next, we examined how cells lacking or expressing SHP2 differ specifically in response to EGF stimulation. SHP2^{WT}-transfected cells responded more strongly to EGF stimulation at the transcriptional level than SHP2^{KO} cells (Fig. 2E and table S2), with changes in gene expression being largely distinct upon EGF stimulation (fig. S2, F and G). Gene set enrichment analysis (GSEA) revealed SHP2^{WT}-dependent changes in expression of genes involved in proliferative signaling, such as the hallmark (*54*) mTORC1 and MYC pathways (Fig. 2F). We also observed an enrichment for genes associated with oxidative phosphorylation and mitochondrial protein degradation pathways (Fig. 2F).

Several early response genes were not significantly differentially expressed as a function of EGF stimulation for SHP2^{WT}-expressing cells. Rather, these genes maintain a high basal expression in SHP2^{WT} cells relative to SHP2^{KO} cells, irrespective of stimulation, suggesting that the mere presence of SHP2 produces some basal level of Ras/MAPK signaling (fig. S2H and table S2). To investigate this further, we determined four unbiased EGF-responsive gene modules based on shared expression patterns across EGF concentrations (Fig. 2G; fig. S2, I and J; and table S2). In SHP2^{WT} cells, at any concentration of EGF, modules 1 and 4 were up-regulated and included EGF response genes (1: *ETV4*/*7*, *CDK4*, *RHOD*, and *PIK3R1*; 4: *EGR1*, *E2F4*, *ETF1*, *POLG*, and *BRD1*) (table S2). By contrast, module 2 and 3, which were only down-regulated in EGF-stimulated SHP2^{WT} cells, contained several tumor suppressors, such as *NRG1*, *MAP3K1*, *CAVIN3*, *EPHA3*/*7*, *CTNNA1*/*3*, and *NOTCH3*. This analysis reveals a broad set of genes coregulated with SHP2^{WT}, but not SHP2^{KO} cells, and highlights the ability of SHP2 to sustain certain EGF signaling markers even without stimulation.

Pathogenic mutations in SHP2 produce unique gene expression profiles

Having established the transcriptional profile of SHP2^{WT} with and without EGF stimulation, we next examined SHP2 mutant profiles, selecting mutations linked to diverse clinical phenotypes and with varying effects on SHP2 structure (Fig. 3A and table S3). Two

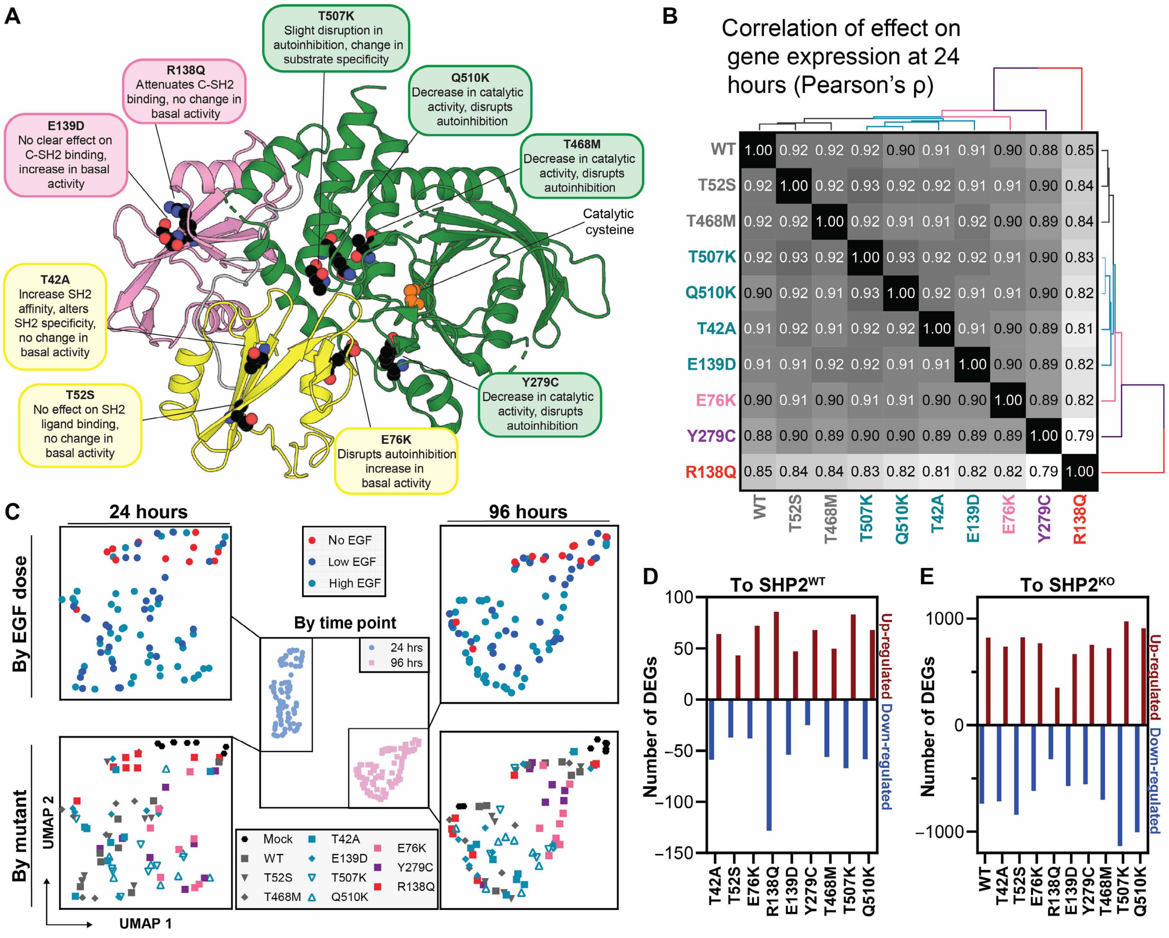

Fig. 3. Transcriptomic profiling of SHP2 variants reveals mutational differences. (**A**) Overview of mutants studied in this screen and their position on the protein. Additional descriptions of the mutants are given in table S3. (**B**) Heatmap of β coefficient correlation (Pearson's ρ) with unsupervised hierarchical clustering, comparing SHP2^{WT} and all SHP2 variants at 24 hours. (**C**) Pseudo-bulked $\log_2$ fold-change expression of cells grouped by time point, SHP2 variant (or mock-transfected cells), and EGF dose, against unstimulated SHP2^{KO} cells. Genes were filtered to the union of DEGs across all mutants (5209 genes). UMAP (*59*) dimensions for 24 hours (hrs., left) and 96 hours (right). Colors of each mutant represent DEG correlation cluster at 24 hours, as seen in (B). (**D**) Number of DEGs per SHP2 variant, compared to SHP2^{KO}, at 24 hours. (**E**) The same as (D), but each SHP2 mutant compared to SHP2^{WT}.

mutations associated with NS were included: Thr42→Ala (T42A), which alters N-SH2–binding affinity and specificity (*27*, *55*), and Glu139→Asp (E139D), a C-SH2 mutation that enhances SHP2 basal catalytic activity but does not appear to affect C-SH2–binding specificity (*26*, *27*). Notably, the E139D mutation has also been found in syndromic JMML (*28*). Glu76→Lys (E76K), which substantially disrupts autoinhibition, and Thr52→Ser (T52S), which modestly affects the N-SH2 ligand–binding pocket, were also included as JMML mutations (*27*, *56*). We included NSML mutations Tyr279→Cys (Y279C) and Thr468→Met (T468M), which reduce catalytic efficiency while increasing SH2 domain accessibility (*13*, *32*, *57*). Last, we included the relatively uncharacterized ALL mutation Q510K, the R138Q mutation found in melanoma and other cancers, which ablates C-SH2–binding capability, and T507K, which disrupts autoinhibition, alters substrate specificity, and has been observed in neuroblastoma, glioblastoma, and hepatocellular carcinoma (*4*–*6*, *27*, *58*).

As with $SHP2^{WT}$, the mutants were expressed in $SHP2^{KO}$ HEK 293 cells, stimulated with a range of EGF concentrations, and harvested at 24 and 96 hours (Fig. 2A and fig. S1A). We performed analysis of differentially expressed genes (DEGs) for each SHP2 variant compared with $SHP2^{KO}$. Of note, *PTPN11* was detected among the highest up-regulated DEGs for all SHP2 variants. To carefully account for the effect of transfection efficiency on SHP2 variant–dependent effect size magnitudes, we performed pair-wise correlation between mutants to assess variant-variant relationships. Overall, there is a strong correlation in gene expression changes for all SHP2 variants at respective time points (Fig. 3B, fig. S3A, and table S3). $SHP2^{R138Q}$ was the most distinct mutant at 24 hours, but even this mutant showed a high correlation of effect on gene expression with $SHP2^{WT}$ (Pearson's ρ of 0.85). At 96 hours, both $SHP2^{R138Q}$ and $SHP2^{Y279C}$ were most distinct (Pearson's ρ of 0.64 and 0.59 with $SHP2^{WT}$, respectively). To visualize both SHP2 variant and EGF dose as variables, we pseudo-bulked (aggregated) the gene expression profiles of cells by time point, SHP2 variant (or mock-transfection), EGF dose, and replicate, for the union of SHP2 variant–dependent DEGs. We calculated the $\log_2$ fold changes to unstimulated $SHP2^{KO}$ cells and initialized a Uniform Manifold Approximation and Projection (UMAP) (*59*) embedding with the resulting $\log_2$ fold changes (Fig. 3C). Consistent with the large number of DEGs across mutants up-regulated and down-regulated (3719 and 3761, respectively, FDR < 0.05) due to stimulation time, time point appears to be the largest determinant of gene expression (Fig. 3C, middle). Within each time point, we observed a loose gradient of EGF dose (Fig. 3C, top), and separation of SHP2 variants (Fig. 3C, bottom, and fig. S3B).

We next determined a common SHP2-dependent transcriptome, which we defined as genes that are differentially expressed compared to $SHP2^{KO}$ cells (β coefficient < −0.05 or > 0.05, FDR < 0.05), shared between at least 5 of 10 SHP2 variants in our study, and not identified as a DEG for our transfection control (fig. S3, C and D). GSEA of this common transcriptome showed overlap with the previously defined gene sets associated with $SHP2^{WT}$ activity, signifying that different SHP2 mutants drive similar transcriptional programs to $SHP2^{WT}$ and each other (fig. S2, A and E, and tables S1 and S3).

Next, we aimed to isolate mutation-dependent changes in transcription. We inspected the top DEGs between $SHP2^{WT}$ cells and SHP2 mutant cells. We identified between 80 and 214 DEGs across all SHP2 mutants at 24 hours (Fig. 3D). $SHP2^{R138Q}$ displayed the largest transcriptional differences compared to $SHP2^{WT}$-expressing cells but was transcriptionally more similar to $SHP2^{KO}$ cells compared to all other tested SHP2 variants (Fig. 3E), suggesting a possible hypomorphic effect at the level of transcription for this SHP2 variant. Consistent with this observation, the pseudobulk profile of the union of SHP2 variant–dependent DEGs for $SHP2^{R138Q}$ clustered separately from $SHP2^{WT}$ and the other variants (fig. S3E).

At 96 hours poststimulation, the number of DEGs is smaller for both comparison to $SHP2^{WT}$ and to $SHP2^{KO}$ (fig. S3, F and G), indicating a return to basal state after stimulation or reduction in SHP2 transient transfection. Together, our initial analysis demonstrates that SHP2 variants impart similar transcriptional profiles but that differences can be detected.

EGF-response dynamics are differentially altered by SHP2 mutations

To isolate SHP2 variant specific differences, we leveraged multiresolution variational interference (MrVI), a deep generative model that performs sample stratification at single-cell resolution while accounting for technical variability (*60*). We recently used MrVI to classify chemical perturbations by their induced transcriptional effects (*42*), and in this study, we applied the model to detect cell state changes imparted by distinct SHP2 variants and EGF stimulation (Fig. 4A). We used UMAP (*59*) for dimension reduction and visualization of cells of the resulting MrVI SHP2 variant/EGF-specific latent space (*Z*-space). $SHP2^{KO}$ and mock-transfected cells form a distinct cluster separate from all SHP2-containing cells, representing a large driver of variation in our model and further demonstrating the impact that SHP2 presence has on gene expression (fig. S4A). In addition, the model appeared to overcome variation due to transfection efficiency (fig. S4B), as *PTPN11* expression was distributed relatively evenly in clusters apart from $SHP2^{KO}$ and mock-transfected cells. To explore more subtle variant specific phenotypes, we visualized cells in the generated MrVI *Z*-space omitting $SHP2^{KO}$ control cells.

We noted a separation in the latent space between cells stimulated with no EGF, low concentration of EGF, or high concentrations of EGF (Fig. 4B). Next, we used Leiden-based community detection (*61*) to cluster SHP2 variant–expressing cells, resulting in five distinct clusters (Fig. 4C and fig. S4C). Analysis of each mutant's distribution across these clusters revealed that $SHP2^{R138Q}$ is predominantly present in cluster 1 at any EGF concentration, whereas other SHP2 variants only appear in this cluster in the absence of EGF stimulation (Fig. 4, D and E, and fig. S4, D and E). Furthermore, $SHP2^{R138Q}$ and $SHP2^{T468M}$ are never present in cluster 5, and $SHP2^{WT}$ and several SHP2 variants ($SHP2^{T42A}$, $SHP2^{T52S}$, $SHP2^{E139D}$, and $SHP2^{Y279C}$) only appear in cluster 5 when stimulated with high doses of EGF (Fig. 4D and fig. S4E). By contrast, $SHP2^{E76K}$, $SHP2^{T507K}$, and $SHP2^{Q510K}$ already populate cluster 5 at low EGF doses. Similar trends were observed when analyzing the counterfactual cell distances determined by MrVI, comparing each mutant and stimulation condition to unstimulated $SHP2^{WT}$ cells (fig. S4F). These observations demonstrate how different structural perturbations to SHP2 can alter its EGF responsiveness.

One notable conclusion from our comparison of SHP2 mutants is that $SHP2^{R138Q}$-expressing cells at any dose of EGF behave most similarly to unstimulated cells expressing almost any other SHP2 variant. Analysis of the DEGs between $SHP2^{WT}$ and $SHP2^{R138Q}$ revealed that $SHP2^{R138Q}$ cells do not express canonical EGF-response genes, such as *EGR1/3*, to the same extent as $SHP2^{WT}$ cells (Fig. 4F). We previously showed that $SHP2^{R138Q}$ has an almost nonfunctional C-SH2 domain (*27*), and colocalized proteins are less likely to be tyrosine-phosphorylated

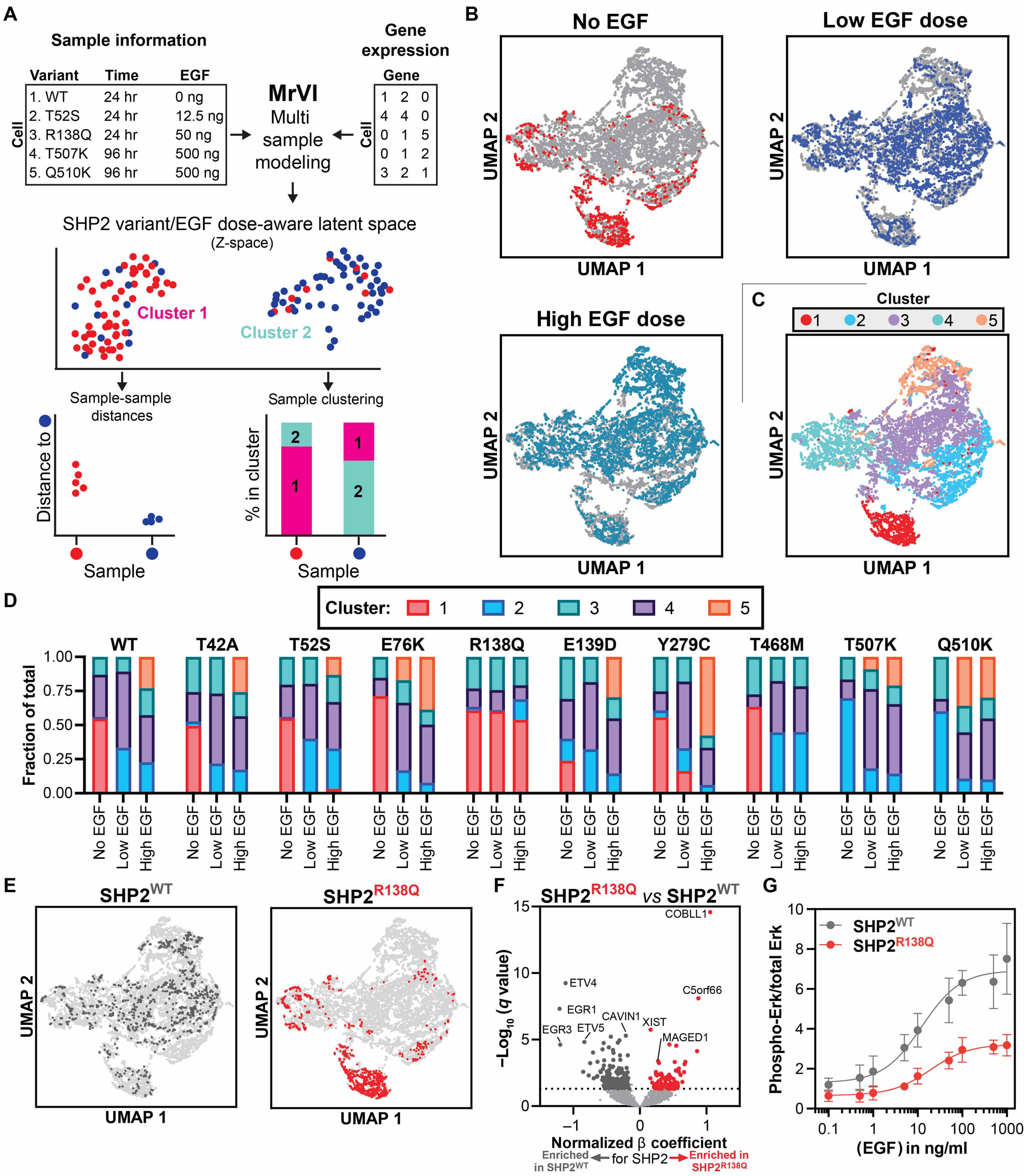

Fig. 4. Cellular response to EGF is altered by WT and mutant SHP2. (**A**) Overview of MrVI. MrVI model was trained on our 24 hours dataset, in which each combination of SHP2 variant and EGF-dose is defined as the sample of origin (96 unique samples), and replicate defined as the technical factor (two unique replicates). (**B**) UMAP of MrVI *Z*-space for all single cells (light gray), excluding $SHP2^{KO}$ cells for visualization. Full MrVI *Z*-space can be found in fig. S4A. Each respective EGF dose group is indicated per UMAP. (**C**) UMAP of MrVI *Z*-space for all single cells, excluding $SHP2^{KO}$ cells. Colors indicate clusters as identified by Leiden community detection. (**D**) Bar plots for each SHP2 mutant and their distribution across clusters. (**E**) UMAPs of the MrVI *Z*-space for $SHP2^{WT}$ and $SHP2^{R138Q}$. (**F**) Volcano plot showing DEGs between $SHP2^{R138Q}$ and $SHP2^{WT}$ across EGF-concentrations. Significant genes (normalized effect size < −0.15 or > 0.15, FDR < 0.05) are labeled in dark gray ($SHP2^{WT}$) and red ($SHP2^{R138Q}$). (**G**) Dose response curves show reduced EGF-response of $SHP2^{R138Q}$ compared to $SHP2^{WT}$. Data points and error bars represent the means and SD from three independent transfections, stimulation, and blotting experiments.

when compared with SHP2^{WT}-colocalized proteins (*34*). C-SH2/phosphoprotein interactions play an important role in localizing SHP2 to signaling complexes (*30*), and SHP2^{R138Q} may thus be unable to interact with EGF receptor (EGFR) pathway phosphoproteins, thereby decreasing responsiveness to EGF stimulation. Consistent with this, EGF-induced changes in gene expression with SHP2^{R138Q} are much smaller than with SHP2^{WT} and do not include canonical EGF response genes (fig. S4G). Furthermore, when we examined Erk phosphorylation as a marker of EGF signaling, we observed a reduced EGF-dependent phospho-Erk levels in SHP2^{R138Q}-expressing cells when compared to SHP2^{WT}-expressing cells, with a modest shift in median effective concentration (EC_{50}) for EGF and large reduction in signal amplitude (Fig. 4G and fig. S4H).

Some of the proliferative, EGF-response genes that were depleted with SHP2^{R138Q} expression, such as *EGR1* and *ETV4*, are also known cancer-associated genes (*62*). To understand which genes might drive the oncogenicity associated with the R138Q mutation, we compared our gene expression profile to genes known to be associated with cancers where SHP2^{R138Q} has been observed, including melanoma and prostatic adenocarcinoma (*62*). *XIST*, a known regulator of malignant melanoma, was significantly enriched in the SHP2^{R138Q} transcriptome (*63*), as was *C5orf66*, a long noncoding RNA, which can function as both an oncogene and tumor suppressor dependent on tissue type (*64*–*67*). We also identified *MAGED1* (melanoma-associated antigen 1), a member of the MAGE family, which is frequently up-regulated in melanoma and other cancers and is a therapeutic target (*68*). However, overexpression of *MAGED1* can suppress cell cycle progression and tissue invasion in other cell systems (*69*). The most up-regulated gene in SHP2^{R138Q}-expressing cells, considering both SHP2-driven effects and EGF-induced effects, was *COBLL1* (Fig. 4F and fig. S4G), which is involved in the oncogenesis of prostate cancer and chronic lymphocytic leukemia (*70*, *71*). Thus, while the SHP2^{R138Q} mutant has a severely attenuated response to EGFR activation, its expression can still up-regulate known oncogenes.

SHP2^{T507K} and SHP2^{Q510K} drive Ras/MAPK signaling in unstimulated cells

Whereas SHP2^{R138Q} was unique in the extent to which it attenuates EGF responsiveness (Fig. 4, E and F), two mutations on the catalytic Q loop (fig. S5A), SHP2^{T507K} and SHP2^{Q510K}, were unique from all other SHP2 variants in that they did not occupy cluster 1 in the absence of EGF stimulation (Fig. 4D and Fig. 5A). Instead, these variants appear to drive an altered basal cellular state that is most represented by cluster 2 (Fig. 4D). The T507K mutation, which has been biochemically characterized (*58*), is associated with several solid tumors, including hepatocellular carcinoma, glioblastoma, and neuroblastoma (*4*–*6*). By contrast, the relatively unstudied Q510K is mainly associated with ALL, although it has also been observed in solid tumors (*72*).

We hypothesized that these mutations converge in their gene expression profiles due to a shared molecular mechanism (Fig. 5B). Specifically, SHP2^{T507K} is known to have reduced catalytic activity against many phosphopeptide substrates; however, because of the introduction of a positive charge in the substrate-binding pocket, SHP2^{T507K} has higher than wild-type (WT) level catalytic efficiency for substrates with a complementary acidic residue, such as Sprouty1 pY53 (*58*). This change in substrate preferences has been linked to T507K-pathogenic signaling, as Sprouty1 is a negative regulator of Ras, and its dephosphorylation by SHP2 causes activation of the Ras/MAPK pathway (*58*). Furthermore, T507K modestly destabilizes the autoinhibited state of SHP2, enhancing its propensity for activation by phosphoprotein binding (*58*). Q510 is a key catalytic residue, and mutations at this site, including Q510K, impair catalysis (*25*, *29*). However, structural models suggest that SHP2^{T507K} and SHP2^{Q510K} might have similarly remodeled active site electrostatics, which could result in similar changes in substrate specificity (Fig. 5C) (*73*).

To test whether T507K and Q510K dysregulate SHP2 through similar molecular mechanisms, we measured the catalytic activities of full-length and isolated PTP domain constructs of SHP2^{WT}, SHP2^{T507K}, and SHP2^{Q510K}. Full-length SHP2^{WT} and SHP2^{Q510K} have comparable activity against the fluorogenic model substrate DiFMUP, while SHP2^{T507K} shows an increase in catalytic efficiency, consistent with a previous study on SHP2^{T507K} (Fig. 5D) (*58*). For the isolated PTP domains, we found that PTP^{Q510K} was substantially less active than PTP^{WT} or PTP^{T507K} against DiFMUP (Fig. 5E). This discrepancy between full-length and PTP proteins could be explained by the ability of the Q510K mutation to disrupt autoinhibition, thereby compensating for the loss of a catalytic residue (Fig. 5B). Differential scanning fluorimetry demonstrated that SHP2^{Q510K} had a dramatically lower melting temperature than SHP2^{WT} and SHP2^{T507K}, indicative of a more open conformation (Fig. 5F) (*25*, *27*, *74*). Even in the context of the isolated PTP domain, the Q510K mutation showed a much lower melting temperature than PTP^{WT} (Fig. 5G), suggesting that this mutant not only disrupts autoinhibition in the full-length protein but also intrinsically destabilizes the isolated PTP domain.

Next, we measured the activity of the isolated PTP domains against four peptide substrates: Paxillin pY118, Sprouty1 pY53, Gab1 pY589, and EGFR pY992, which were previously used to profile the change in SHP2^{T507K} substrate preferences (*58*). Consistent with previously reported results, we saw an increase in preference for Sprouty1 for PTP^{T507K} when compared to PTP^{WT} (Fig. 5H and fig. S5B). Notably, while PTP^{Q510K} overall showed strong catalytic impairment, we observed an increased preference for Sprouty1, suggesting that the Lys substitutions on the two nearby sites have convergent effects on substrate preferences (Fig. 5H and fig. S5B). Furthermore, the destabilizing effect of the Q510K mutation disrupts autoinhibition to such an extent that the full-length protein has comparable activity to SHP2^{WT} (Fig. 5, B and D). While substrates other than Sprouty1 may be at play for either mutant, and other structural explanations might also be relevant, our biochemical and transcriptomic results suggest that the Q510K and T507K operate at least partly through similar mechanisms. Intriguingly, in cells expressing SHP2^{WT} or these mutants, we observed that SHP2^{Q510K} uniquely increased global phosphotyrosine levels across all tested EGF stimulation concentrations (fig. S5C), further demonstrating that this mutant can dysregulate signaling.

The identity of the Q510 substitution fine-tunes functional outcomes

In addition to T507K and Q510K, there are several other pathogenic mutations in the Q loop of SHP2 (fig. S5A). In particular, Q510 has several other known pathogenic substitutions (Gln510→His/Glu/Leu/Pro/Arg; Q510H/E/L/P/R), which can have distinct disease outcomes (table S3). With the exception of Q510K, all observed Q510 mutations are associated with NSML, with Q510L/P/R also having been implicated in NS. Like Q510K, Q510E is also associated with ALL, whereas Q510P, L, and H have been found in patients with AML. In addition, all Q510 mutants occur in solid cancers. While loss of the WT residue may fully explain dysregulatory effects

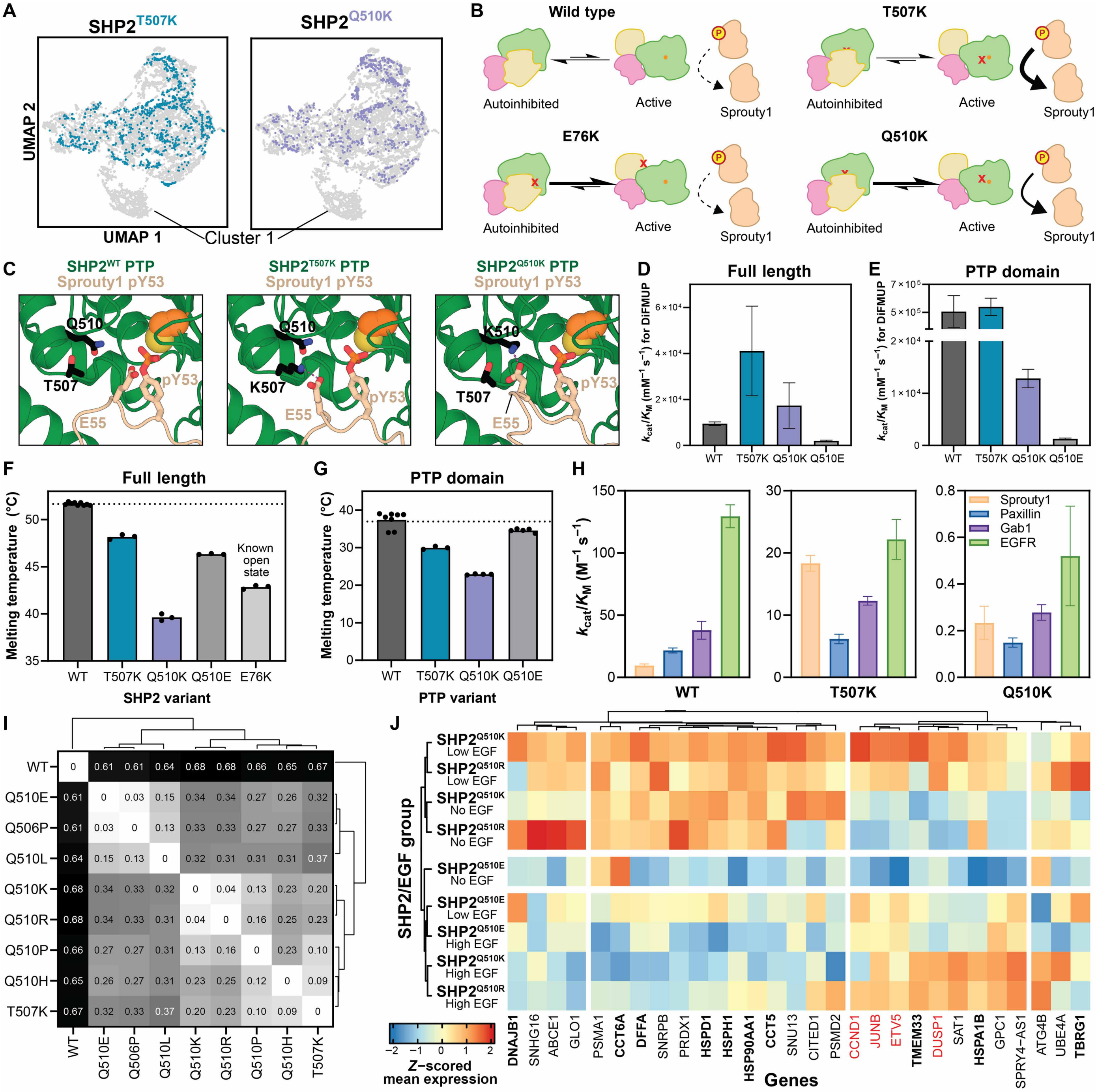

Fig. 5. Q-loop mutations can alter substrate specificity and conformational stability to modulate downstream transcription. (**A**) UMAP of the MrVI *Z*-space for SHP2^{T507K} and SHP2^{Q510K} shows absence of cells in cluster 1. (**B**) Schematic showing the structure and activity changes in SHP2^{Q510K} relative to SHP2^{WT}, SHP2^{E76K}, and SHP2^{T507K}. The Q510K mutation shifts the protein toward the open conformation, while also enhancing Sprouty1 dephosphorylation. (**C**) AlphaFold 3 models of SHP2^{T507K} (top) and SHP2^{Q510K} (bottom), bound to Y53-phosphorylated Sprouty1 in the active site, showing the proximity of K507 and K510 with E55 on Sprouty1. (**D**) Catalytic efficiencies of full-length SHP2^{WT}, SHP2^{T507K}, and SHP2^{Q510K} against DiFMUP. (**E**) The same as (D), but for the isolated PTP domains. (**F**) Melting temperatures for full-length SHP2^{WT}, SHP2^{T507K}, SHP2^{Q510K}, and SHP2^{Q510E}. SHP2^{E76K}, a known open conformation mutant, is shown for reference. (**G**) The same as (F), but for isolated PTP domains. (**H**) Dephosphorylation assay with PTP^{WT}, PTP^{T507K}, and PTP^{Q510K} showing switch in substrate preferences. Full peptide sequences are indicated in Materials and Methods. (**I**) Pairwise MrVI counterfactual cell distances, showing the largest distances between SHP2^{WT} and any Q-loop mutant. SHP2^{Q510K} and SHP2^{Q510R} show the smallest distance observed. (**J**) Heatmap for DEGs for SHP2$^{Q510K/R}$ versus SHP2^{WT}. *Z*-scored mean expression for SHP2^{Q510K}, SHP2^{Q510R}, and SHP2^{Q510E} are visualized. Gene names in bold represent chaperone and protein folding genes. Red gene names represent EGF response genes.

of mutations at a particular site, evidence from other proteins such as Ras GTPases suggests that the identity of the substituted amino acid can also dictate functional outcomes (*75*, *76*). For SHP2, we see differences in basal activity and stability for Q510K and Q510E that could have downstream consequences (Fig. 5, D to G). Thus, we conducted another transcriptomic screen with an additional six SHP2 variants focused on Q-loop mutations, including all disease-relevant Q510 substitutions, T507K, and another common mutation at a catalytic residue, Q506P (fig. S6, A to D). All Q-loop mutants appeared distinct from SHP2^{WT} and induced a similar number of DEGs relative to SHP2^{WT} (fig. S6, E and F).

Building on the observation from our previous screen that SHP2^{T507K} and SHP2^{Q510K} were most distinct from other SHP2 variants in unstimulated conditions, we trained a specific MrVI model on the unstimulated cells at 24 hours. Then, we calculated the counterfactual cell distances between different SHP2 variants in this screen. Consistent with the trend found in our correlation of β coefficients (fig. S6E), we found that SHP2^{WT} shows the largest distance to any of the mutants in our screen (distance = 0.61 to 0.68) (Fig. 5I). Furthermore, we found that SHP2^{Q510K} and SHP2^{Q510R}, which introduce a positive charge, are remarkably similar (distance = 0.04), whereas SHP2^{Q510E}, which brings about a negative charge, is the most distant mutant from SHP2$^{Q510K/R}$ (distance = 0.34) (Fig. 5I).

To understand what drives these trends, we analyzed DEGs between SHP2^{WT} and the group of SHP2$^{Q510K/R}$. We identified several EGF response genes, such as *JUNB*, *CCND1*, *DUSP1*, and *ETV5*, as increasingly expressed in cells with the SHP2$^{Q510K/R}$, many of which were not up-regulated to the same extent with SHP2^{Q510E} (Fig. 5J, red genes, and table S3). This suggests that SHP2^{Q510R} potentially shares the altered cell state that we previously observed for SHP2^{Q510K} and SHP2^{T507K}, which could be explained by a similar charge-based change in substrate specificity. By contrast, SHP2^{Q510E} is effectively catalytically dead but has a mildly destabilized autoinhibited state, somewhere between SHP2^{WT} and SHP2^{Q510K} (Fig. 5, D to G). Thus, it can only drive signaling through its scaffolding functions. Notably, SHP2^{Q510E} is most similar to SHP2^{Q506P} in our transcriptomics data (Fig. 5I). This mutation also reduces catalytic activity, and it has been reported to destabilize SHP2 autoinhibition to the same extent as SHP2^{Q510E} (*29*).

Last, we were surprised to find that the intersection of the SHP2^{Q510K} and SHP2^{Q510R} data showed enrichment for genes encoding chaperones and other proteostasis machinery (table S3) (*77*). We compared the *Z*-scored mean expression of these identified genes in SHP2$^{Q510K/R}$ data to SHP2^{Q510E} data and found that these genes are expressed to a much lesser degree in SHP2^{Q510E} (Fig. 5J, bolded genes). One plausible explanation for this difference is that the Q510R mutation, similar to Q510K, may destabilize the PTP domain of SHP2, triggering proteostasis machinery. By contrast, Q510E is much less destabilizing, both for full-length SHP2 and the isolated PTP domain (Fig. 5, F and G), which may explain why a similar response is not observed in SHP2^{Q510E}-expressing cells. Overall, our data suggest that the distinct substitutions at Q510 can have diverse effects on protein conformation, stability, and activity, which are likely to shape unique downstream signaling and transcriptional programs.

DISCUSSION

In this study, we conducted two multiplexed single-cell transcriptomic screens with cells expressing a variety of pathogenic SHP2 mutants, stimulated with a range of EGF doses across multiple time points. First, by comparing SHP2^{WT}-expressing cells directly to SHP2^{KO} cells, we show that the presence of SHP2 is essential for expression of EGF-response genes, such as *EGR1/3* and *ETV4/5*. As a result, SHP2^{KO} cells are defective in manifesting a cellular response to EGF stimulation. SHP2^{WT}-expressing cells showed up-regulation of several key cell signaling pathways, including mTORC1 and MYC pathways. The SHP2^{KO} cells only showed up-regulation of fibroblast growth factor 3 (FGFR3)–related signaling, which is consistent with previous studies showing that SHP2 inhibition led to compensatory activation of FGFR signaling and rebound ERK activity, suggesting that cells navigate the absence of SHP2 by escaping to other cell signaling pathways (*78*, *79*).

Next, we compared SHP2^{WT} to nine SHP2 mutants, chosen for their range of effects on protein structure and activity, along with diverse disease contexts. We observed a distinctive correlation between the protein-level mechanism of dysregulation and resulting cell states. For example, SHP2^{R138Q}, which has a defective C-SH2 domain (*27*), attenuated proximal signaling in response to EGF (lower Erk phosphorylation) and led to notably diminished transcription of EGF-response genes. SHP2^{R138Q} retains normal catalytic activity, both in basal conditions as well as when the N-SH2 domain is engaged by a phosphopeptide (*27*), illustrating how noncatalytic properties of SHP2 are critical for EGF signaling. Previous work has shown that catalytically dead SHP2^{C459S} is unable to activate the Ras/MAPK pathway in response to EGF stimulation (*29*). This suggests that both the C-SH2–binding function and PTP domain catalytic activity of SHP2 are needed for activation of the Ras/MAPK pathway in response to EGF stimulation, highlighting the importance of the scaffolding function of SHP2, as well as its interplay with catalytic activity. Our transcriptomics data show that the gene expression profile mediated by SHP2^{R138Q} is distinct from SHP2^{KO}, indicating that some of its signaling functions are intact, and specific oncogenes are differentially overexpressed compared to SHP2^{KO} or SHP2^{WT}.

Our transcriptomics data also revealed unexpected instances of functional convergence by two apparently unrelated mutations and functional divergence by different substitutions at the same site. Specifically, we found that the unstudied mutant SHP2^{Q510K}, identified in cancer, partly phenocopies SHP2^{T507K} by introducing a lysine into the substrate-binding pocket of the PTP domain and altering substrate specificity. As a result, both of these mutants mediate similar EGF-dependent transcriptional responses that are distinct from all other mutants in our screen. We observed a substantial increase in global phosphotyrosine levels in the presence of SHP2^{Q510K}, compared to SHP2^{WT} and even compared to SHP2^{T507K}. We speculate that this mutant can strongly activate a tyrosine kinase, causing an overall increase in phosphorylation of cellular proteins. Several studies have shown that SHP2 can activate c-Src through a noncatalytic binding interaction and that this interaction happens more readily for open-conformation SHP2 mutants (*80*–*82*). Our data suggest that the autoinhibited state of SHP2 is substantially destabilized by the Q510K mutation. This would put SHP2^{Q510K} in an open conformation that is competent to potently activate c-Src, providing a plausible mechanism through which this mutant could aberrantly increase phosphotyrosine levels in the cell.

Other pathogenic mutations at Q510 unexpectedly showed a diverse range of downstream effects. SHP2^{Q510K} and SHP2^{Q510R}, which both introduce a positive charge in the active site, produce the most similar gene expression profiles, whereas we found that SHP2^{Q510E}, which

introduces a negative charge, has distinct effects on protein stability, autoinhibition, and catalytic activity, resulting in a divergent transcriptome. For many known missense mutations in SHP2, it appears that the loss of the original residue is more detrimental to protein function than the identity of the resulting amino acid. For example, while $SHP2^{E76K}$ is well-known and well-studied, substitutions to D, G, A, Q, V, and M at this position have also been identified in patients (*72*), and all of these substitutions disrupt autoinhibition to hyperactivate SHP2 (*25*, *28*, *72*). By contrast, for Q510, we find that the identity of the resulting mutation at a site can also dictate downstream functions. This "original-centric" view of pathogenic mutations is being challenged in other systems as well, including the well-studied oncogene Ras, where different G12 mutations have distinct effects on protein interactions and guanosine triphosphate hydrolysis rates (*75*, *76*).

A critical feature of our experimental design that yielded the aforementioned insights is that we conducted these screens with transient expression of variants in a homogenous genetic background, $SHP2^{KO}$ HEK 293 cells. This approach isolates how structural and biochemical consequences of mutations in SHP2 propagate to changes in cell state, without other confounding factors, including genetic, transcriptomic, or proteomic variation. Through this approach, we were able to amplify mutant-specific outcomes and connect changes in cell state to nuanced mutant-dependent changes in SHP2 structure, stability, and molecular recognition. We acknowledge that the mutants in our study occur in a broad range of human diseases and thus affect a broad range of cell types. In the congenital disorders NS and NSML, SHP2 mutations are inherited and systemic, whereas somatic SHP2 mutations in cancers are localized to specific cell and tissue types. Thus, these mutations naturally drive diseases in a wide array of cellular and mutational contexts. Our reductionist approach provides a baseline for connecting SHP2 structural perturbations to cellular outcomes and lays the foundation for deeper mechanistic studies in disease-relevant cell lines, animal models, or patient samples.

In our study, we focused on just over a dozen pathogenic mutations that have varied effects on SHP2 at the molecular level. By analyzing how expression of these diverse mutants influenced the transcriptome, we were able to show that corresponding changes to the conformational state of SHP2, to noncatalytic protein-protein interactions, and even to its substrate specificity, can propagate into major differences in cellular outcomes, separate from effects coming from basal catalytic activity. Our results highlight the bidirectional value of integrating structural and cellular data: Consideration of protein structure and biochemistry can inspire insightful cellular experiments, while unbiased assessments of cellular phenotypic effects—such as through transcriptomics—can, in turn, reveal unexpected biochemical insights. In the future, one can envision taking a more expansive and unbiased approach to gain even deeper insights. The analysis of comprehensive scanning mutagenesis libraries, coupled with functional selection and deep sequencing, is yielding new insights into protein stability, regulation, molecular recognition, catalysis, and drug resistance (*83*). Combining these deep mutational scanning approaches with multiplexed single-cell transcriptomics could yield a powerful framework for mapping the effects of mutations from the molecular to the cellular scale.

MATERIALS AND METHODS

Cell culture

Cells were cultured in a 37°C tissue culture incubator with 5% CO_2. Cells were discarded by passage 25 and tested for mycoplasma every 3 months. HEK 293 cells were grown in Dulbecco's modified Eagle's medium (DMEM) with 10% fetal bovine serum (FBS) and 1% penicillin-streptomycin. HEK 293 SHP2 KO cells were grown in DMEM with 10% FBS. Human EGF was purchased in lyophilized form (#E9644, Sigma-Aldrich) and reconstituted in 10 mM acetic acid.

Transfection and EGF stimulation for scRNA sequencing

HEK 293 $SHP2^{KO}$ (10^6) cells were seeded in a six-well plate, and transfected 24 hours later with 2 μg of DNA in 200 μl of empty DMEM using 6 μl of polyethylenimine (PEI). Early the next morning, each well was trypsinized and reseeded into a 96-well plate to a final density of 10,000 cells per well. Twelve hours after reseeding, the cells were checked for adherence, and culture medium was aspirated. Cells were serum-starved overnight in DMEM + 0.2% FBS. After 12 hours of serum starvation, EGF was added to the cells in DMEM + 0.2% FBS to a final concentration of 1000, 500, 250, 100, 50, 25, 12.5, and 0 ng/ml (unstimulated). At 24 and 96 hours, the cells were harvested and nuclei were hashed and frozen at −80°C until further processing. Replicates refer to two separate 96-well plates containing identical SHP2 variant groups, receiving EGF stimulation independently, with altered plate layout to account for position effects. The number of unique conditions for the first screen totals 192 as cells from 12 SHP2 variants (9 mutants, WT, KO, and mock-transfected control) × 8 EGF concentrations (0 to 1000 ng/ml) × 2 time points (24 and 96 hours post–EGF stimulation) were measured in replicate. The second screen, independent of the preliminary screen, was represented by 10 SHP2 variants (8 mutants, WT, and KO) × 8 EGF concentrations (0 to 1000 ng/ml) × 2 time points (24 and 96 hours post–EGF stimulation) measured in replicate. In total, 15 unique mutants were profiled over two screens: 9 from the initial and 6 from the Q-loop screen.

EGF dose-response curves

For Erk/p-Erk dose-response curves for $SHP2^{WT}$ and $SHP2^{R138Q}$, 5 × 10^6 HEK 293 $SHP2^{KO}$ cells were seeded in a 15-cm plate in full DMEM. The next day, the cells were transfected with 20 μg of DNA in 2 ml of empty DMEM using 60 μl of PEI. In the morning, transfection medium was replaced with full DMEM; 12 hours later, the cells were serum-starved in DMEM + 0.2% FBS. The next morning, the cells were harvested in phosphate-buffered saline (PBS) by scraping, washed two times in 1 ml of PBS, and split into separate tubes corresponding with the number of concentrations. Pellets were preheated at 37°C for 2 min before stimulation. Cells were stimulated with EGF (0 to 1000 ng/ml) in preheated PBS on a 37°C heat block. After exactly 2 min, tubes were placed on ice and spun down in a refrigerated tabletop centrifuge (5 min, 1000*g*). Supernatant was aspirated, and pellets were snap-frozen and stored in −80°C until further processing. The cells were lysed in 100 μl of lysis buffer [20 mM tris-HCl (pH 8.0), 137 mM NaCl, 2 mM EDTA, 10% glycerol, and 0.5% NP-40 + protease inhibitors + PTP inhibitors] for 30 min on ice. The cells were spun at 17.7 rpm for 15 min at 4°C. The supernatant was transferred to a clean Eppendorf tube and stored at −20°C.

Protein concentration was determined using a bicinchoninic acid (BCA) assay, and absorbance was measured at 562 nm using a BioTek Synergy Neo2 multimode reader. Fifteen micrograms of protein was loaded onto a gel. As not all samples fit on one gel, we ran six samples (three EGF concentrations) on both gels. Gels were transferred onto a nitrocellulose membrane using TurboBlot (Bio-Rad), and the membrane was blocked using 5% bovine serum albumin (BSA) in

tris-buffered saline (TBS) for 1 hour at room temperature. Membranes were rinsed with TBS with 0.1% Tween-20 (TBS-T) and incubated with primary antibodies in TBST + 5% BSA overnight at 4°C. The following antibodies and dilutions were used: Erk1/2 (Cell Signaling Technology, #4696S), 1:2000; p-Erk1/2 (Cell Signaling Technology, #4377S), 1:1000; Vinculin (Cell Signaling Technology, #13901S), 1:1000; and Myc (Invitrogen, # R95025), 1:5000. Membranes were washed and incubated with secondary antibodies (IRDye 680RD goat anti-rabbit immunoglobulin G (IgG), LiCor, #926-68071; and IRDye 800CW goat anti-mouse IgG, LiCor, #926-32210). Blots were imaged on a LiCor Odyssey, and bands were quantified using ImageStudio. Values of the second gel (higher EGF concentrations) were scaled to gel 1 (lower EGF concentrations) by using the quantified values for the six overlapping samples. Curves were visualized using GraphPad Prism v10.4.0, which was also used to calculate EC_{50} for $SHP2^{WT}$ and $SHP2^{R138Q}$.

For global phosphotyrosine responses for $SHP2^{WT}$, $SHP2^{T507K}$, and $SHP2^{Q510K}$, 3×10^6 $SHP2^{KO}$ HEK293 cells were seeded in a 10-cm plate. The next day, the cells were transfected with 10 μg of DNA in 1 ml of empty DMEM using 30 μl of PEI. In the morning, transfection medium was replaced with full DMEM; 12 hours later, the cells were serum-starved in DMEM + 0.2% FBS. The next morning, the cells were harvested in PBS by scraping, washed two times in 1 ml of PBS and split into separate tubes corresponding with the number of concentrations. Pellets were preheated at 37°C for 2 min before stimulation. The cells were stimulated with EGF (0 to 1000 ng/ml) in preheated PBS on a 37°C heat block. After exactly 2 min, the tubes were placed on ice and spun down in a refrigerated tabletop centrifuge (5 min, 1000*g*). Supernatant was aspirated, and pellets were snap-frozen and stored in −80°C until further processing. The cells were lysed in 100 μl of lysis buffer [20 mM tris-HCl (pH 8.0), 137 mM NaCl, 2 mM EDTA, 10% glycerol, and 0.5% NP-40 + protease inhibitors + PTP inhibitors] for 30 min on ice. The cells were spun at 17.7 rpm for 15 min at 4°C. The supernatant was transferred to a clean Eppendorf tube and stored at −20°C.

Protein concentration was determined using a BCA assay, and absorbance was measured at 562 nm using a BioTek Synergy Neo2 multimode reader. Fifteen micrograms of protein was loaded onto 26-well Criterion gel. Gel was transferred onto a nitrocellulose membrane using TurboBlot (Bio-Rad), and the membrane was blocked using 5% BSA in TBS-T for 1 hour at room temperature. Membranes were rinsed with TBS-T and incubated with primary antibodies in TBST + 5% BSA for 2 hours at room temperature. The following antibodies and dilutions were used: Myc (Invitrogen, # R95025), 1:5000; pTyr (Cell Signaling Technology, 8954S), 1:1000; and glyceraldehyde-3-phosphate dehydrogenase (Sigma-Aldrich, G8795); 1:8000. Membranes were washed and incubated with secondary antibodies (IRDye 680RD goat anti-rabbit IgG, LiCor, #926-68071; and IRDye 800CW goat anti-mouse IgG, LiCor, #926-32210). Blots were imaged on a Bio-Rad ChemiDoc MP.

Nuclei preparation and hashing

For both screens, cells were trypsinized and moved to V-bottom plates at 24 and 96 hours post–EGF exposure. Upon washing with ice-cold 1× PBS, the cells were lysed with EZ Lysis Buffer (Sigma-Aldrich) supplemented with 1% diethyl pyrocarbonate (Sigma-Aldrich), 0.1% Superase RNase Inhibitor (Thermo Fisher Scientific), and 500 fmol of hashing oligo. After lysis, nuclei were fixed with the addition of 1.25% formaldehyde in 1.25× PBS (to a final well concentration of 1% and 1×, respectively) and incubated on ice for 10 min. Nuclei were pooled into a plastic reservoir and moved into a 50-ml conical for centrifugation at 650*g* for 5 min at 4°C. Supernatant was removed from the nuclei pellet, and nuclei were washed once with nuclei suspension buffer [(NSB) 10 mM tris-HCl (pH 7.4), 10 mM NaCl, 3 mM MgCl2, 1% Superase RNA Inhibitor (Thermo Fisher Scientific), and 1% Ultrapure BSA (0.2 mg/ml; New England Biosciences)]. Nuclei were resuspended in NSB, slow-frozen in 10% dimethyl sulfoxide (DMSO), and stored at −80°C until single cell combinatorial indexing RNA sequencing (sci-RNA-seq) library preparation.

Library preparation and sequencing

Hashed nuclei were thawed and subjected to three-level combinatorial indexing protocols adapted from previous methods (*42*, *44*–*46*). Nuclei were spun, resuspended in NSB, and sonicated at low power for 12 s (Bioruptor). Upon counting, 21 μl of nuclei were moved to 96-well low-adhesion polymerase chain reaction (PCR) plates with 2 μl of 10 mM deoxynucleotide triphosphate (dNTP) solution mix [New England Biosciences (NEB)], 2 μl of 100 μM indexed oligo-shortdT primers, 2 μl of 100 μM indexed random hexamer primers, and 14 μl of a reverse transcription master mix consisting of 14.29% 100 mM dithiothreitol, 14.29% 100 mM RNaseOUT Ribonuclease Inhibitor, 57.14% 5× SuperScript IV First-Strand Buffer, and 14.29% SuperScript IV Reverse Transcriptase. Reverse transcription was carried out with an increasing temperature gradient. Post–reverse transcription, nuclei were pooled and distributed as 10 μl into a 96-well plate(s) for ligation steps. Briefly, 8 μl of indexed ligation primers were added to each well, along with a 4.8-μl 3:2 master mix of T4 ligase buffer:T4 ligase (NEB) and 9.4 μl of nuclei buffer with BSA [(NBB) 10 mM tris-HCl (pH 7.4), 10 mM NaCl, 3 mM MgCl2, and 1% Ultrapure BSA (0.2 mg/ml)]. Ligation was carried out at 25°C for 1 hour. The resulting nuclei were pooled, washed with NBB, and distributed as 1500 nuclei in 5 μl of NBB per well, where some plates were stored for future processing. Next, 5 μl of a second strand synthesis mix consisting of 60% elution buffer (Qiagen), 27% second strand synthesis buffer (NEB), and 13% second strand synthesis enzyme mix (NEB) was added, and second strand synthesis was carried out at 16°C for 3 hours. Post–second strand synthesis, tagmentation was performed at 55°C for 5 min after the addition of 1/50 μl per well of N7-adaptor loaded Tn5 and subsequent quenching with DNA binding buffer (Zymo) for 5 min at room temperature. The resulting double-stranded DNA (dsDNA) was purified using a 1× SPRIbead clean-up within the 96-well plate. The dsDNA was eluted in buffer EB and then moved to a clean 96-well PCR plate. To the 16 μl of eluted product, 2 μl of P5 PCR primer and 2 μl of P7 PCR primer were added to wells in an indexed well-specific combination. Further, 20 μl of 2× NEBnext PCR master mix (NEB) was added, and PCR to add the adaptors was carried out. The final PCR product was pooled and subjected to a 0.7× SPRIbead cleanup for library cDNA purification and 1× cleanup for hash fraction purification. Library concentrations were determined by Qubit (Invitrogen) and were visualized by TapeStation DNA D1000. The resulting libraries were sequenced on the Element Biosciences AVITI for the initial SHP2 mutant screen according to the manufacturer's instructions and on the Illumina NovaSeq XPlus (Novogene) for the follow-up SHP2 Q510 screen.

Data preprocessing and generation of count matrix

Raw base call files were obtained from Illumina BaseSpace or AVITI storage and were used to generate fastq files using bcl2fastq v2.20.0.422

or bases2fastq version 1.5.0.962525890, respective to sequencing platform. A custom data processing pipeline, adapted from previous publications (*41*), was used to process fastq data into a single-cell count matrix. First, reverse transcription and ligation barcodes were assigned to reads with a mismatch allowance of 1 base pair, and reads assigned to oligo-shortdT primers were separated from those assigned to random hexamer primers. After index assignment, polyA sequences were trimmed using TrimGalore version 0.6.10 and CutAdapt version 2.6. Upon polyA trimming, the reads were aligned to human GRCh38 using the STAR aligner version 2.7.9a. Aligned reads were filtered for quality, and duplicates and were assigned to genes using bedtools version 2.26.0, as described previously. The resulting unique read assignments from both primers were combined and collapsed by cell and gene, and a celldataset (CDS) object was generated using the raw sparse count matrix, cell annotations, and gene annotations with the R package monocle3. Cell barcodes were determined to be cells upon filtering the CDS by a cutoff of 300 unique molecular identifiers (UMIs), determined visually with the kneeplot of cell rank by UMI count. Last, doublets were detected with scrublet and were filtered based on the doublet score distribution.

In parallel, hash assignments were determined from demultiplexed untrimmed oligo-shortdT reads as described previously (*41*, *47*). Briefly, hash barcodes were assigned to reads with a mismatch allowance of 1 base pair and if the read was adjacent to repeated A sequences, corresponding with hash sequence design. Duplicate hash reads were filtered by UMI and were collapsed into hash assignment counts by cell. Hashes were assigned to cells by two criteria: (i) a cell having ≥5 hash UMIs and (ii) a ratio of the cell's top hash UMI to second best hash UMI of 2.5. The monocle3 package was used to manipulate, batch align, and visualize the resulting data.

SHP2 mutant–specific differential gene expression test

SHP2 variant was annotated in the object colData as a factor to designate either KO or WT as the reference comparison group. DEGs were calculated by fitting expression to a quasi-Poisson regression model of a gene within each time point as a function of SHP2 variant, EGF exposure concentration, and technical replicate using the monocle3 R package fit_models function. The tests were limited to genes expressed in at least 1% of all cells in the experiment. *P* values for each DEG test were FDR corrected (specifically, Benjamini-Hochberg correction for multiple hypotheses). Significant SHP2 variant DEGs were defined as genes where the SHP2 variant coefficient was FDR < 0.01 and a normalized effect (β-coefficient) of >0.05, unless otherwise indicated. SHP2-mutant specific coefficients were calculated using the model below with cells across all EGF concentrations and replicate for each time point

Expression ∼ SHP2_mut + EGF_dose + replicate

EGF-dose specific differential gene expression test

For each SHP2 variant, DEGs were calculated by fitting expression to a quasi-Poisson regression model of a gene as a function of EGF exposure concentration ($\log_{10}$ transformed with a pseudo-count of 0.1), time point, and technical replicate using the monocle3 R package fit_models function. The tests were limited to genes expressed in at least 1% of all cells in the experiment. *P* values for each DEG test were FDR corrected (Benjamini-Hochberg correction for multiple hypotheses). Significant EGF DEGs for each variant were defined as genes where the EGF dose coefficient was FDR < 0.01 and a normalized effect magnitude (β coefficient) of >0.05, unless otherwise indicated. The formula for obtaining EGF dose–specific normalized effects for each mutant is as follows

Expression ∼ EGF_dose + time point + replicate

GSEA of SHP2^{WT} and SHP2^{KO} EGF-driven DEGs

The fgsea R package was used to assess the enrichment of gene sets in EGF-induced DEGs for SHP2^{WT} and SHP2^{KO}. Briefly, for SHP2^{WT} and SHP2^{KO} separately, EGF-induced DEGs were ranked by descending effect (β coefficient) and were tested for enrichment using the fgsea function against gene sets found in Hallmark pathways (h.all.v6.0.symbols) and Reactome pathways (c2.cp.reactome.v2024.1.Hs.symbols), downloaded from MSigDB. MYC-targets v1 was included to allow identification of broad effects, whereas MYC-targets v2 allows for detecting more direct MYC-driven effects. *P* values for each test were FDR corrected (Benjamini-Hochberg correction for multiple hypotheses), and significantly enriched gene sets were designated as FDR <0.05.

Defining SHP2^{WT} and SHP2^{KO} EGF-driven gene modules

Gene modules were defined using the union of EGF-induced DEGs for SHP2^{WT} and SHP2^{KO} (FDR <0.05). Upon filtering the cds object to SHP2^{WT} and SHP2^{KO} cells and EGF-induced DEG union genes, gene modules were defined using the monocle3 R package find_gene_modules function with a resolution of 1×10^{-2}. Gene modules were validated by visual inspection of silhouette plots. For each cell and gene module, expression of genes was aggregated, and the mean expression for each SHP2 variant and EGF exposure group was *z*-scored for visualization with the R package ComplexHeatmap.

SHP2 variant pair-wise correlation coefficient visualization

SHP2 variant pair-wise Pearson correlation coefficients were calculated using the normalized effects (β coefficients) of the union of DEGs across SHP2 variants [FDR < 0.01 and abs(normalized effect) > 0.05]. Unsupervised hierarchical clustering (ward.D2) and visualization of the correlation coefficients was performed using ComplexHeatmap.

MrVI model training

MrVI was downloaded and imported from the publicly available scvi-tools. For the first SHP2 mutant screen, feature selection consisted of taking the union of the top 300 highly variable genes (determined with scanpy) for each time point_SHP2 variant subset of cells, for a total of 3318 genes.

We then trained a MrVI model on all 24-hour post-EGF exposure cells with the sample key defined as unique SHP2 variant, EGF concentration combination and the batch key defined as replicate. For all trained models, we used the recommended default model arguments: n_latent = 30; n_latent_u = 10; qz_nn_flavor = "attention"; px_nn_flavor = "attention"; use_map (qz_kwargs) = True; stop_gradients (qz_kwargs) = False; stop_gradients_mlp (qz_kwargs) = True; dropout_rate (qz_kwargs) = 0.03; stop_gradients (px_kwargs) = False; stop_gradients_mlp (px_kwargs) = True; h_activation (px_kwargs) = "nn.softmax"; low_dim_batch (px_kwargs) = True; dropout_rate (px_kwargs) = 0.03; learn_z_u_prior_scale = False; z_u_prior = True; and u_prior_mixture = False. We used the following training arguments in tandem with the recommended default model arguments: max_epochs = 400; batch_size = 256; early_stopping = True; early_stopping_patience = 15; check_val_every_n_epoch = 1; train_size = 0.9; lr (pl_kwargs) =

2×10^{-3}; n_epochs_kl_warmup (plan_kwargs) = 20; max_norm (plan_kwargs) = 40; eps (plan_kwargs) = 1×10^{-8}; and weight_decay (plan_kwargs) = 1×10^{-8}.

For the follow-up Q510 screen, feature selection (2358 genes) and model training were conducted as with the preliminary screen. Separate models were trained upon filtering cells to respective EGF groups (no EGF, low, and high) to increase resolution to capture differences between mutants within respective EGF exposure groups.

MrVI visualization and SHP2 variant cluster proportion

To visualize the output of the MrVI models, we generated UMAPs for the *U* and *Z* latent spaces. Specifically, the cell by *Z* latent dimensions were exported and added to the cds object in R where a UMAP embedding was generated and visualized using monocle3 reduce_dimensions and plot_cells functions. For downstream analysis, SHP2^{KO} and mock-transfected cells were filtered out to emphasize differences between remaining variants. Leiden community detection (cluster_cells) was performed at a resolution of 1×10^{-3}, and robustness of clustering was determined with silhouette plots. Counts of cells per cluster were used to determine cluster proportions for each SHP2 variant.

The functional relationship between the sample-unaware *U*-space and the sample-aware *Z*-space in MrVI can be used to directly estimate single-cell resolution sample-sample distance. After model training, both single-cell and mean sample-sample counterfactual distances were obtained using the get_local_sample_distances function. Single-cell counterfactual distances were visualized as distributions, and mean sample-sample counterfactual distance matrices were visualized as heatmaps using ComplexHeatmap.

Purification of full-length SHP2 and SHP2 PTP domains

Full-length SHP2 variants were cloned into a pET28-His-TEV plasmid. BL21(DE3) cells were transformed with the respective plasmids and were grown in TB supplemented with kanamycin (100 μg/ml) at 37°C until cells reached an optical density at 600 nm of 0.5. Isopropyl-β-d-thiogalactopyranoside (1 mM) was added to induce protein expression, which was carried out at 18°C overnight. Cells were centrifuged and subsequently resuspended in lysis buffer [50 mM tris (pH 7.5), 300 mM NaCl, 20 mM imidazole, 10% glycerol, and freshly added 2 mM β-mercaptoethanol]. The cells were lysed using sonication (Fisherbrand Sonic Dismembrator), and spun down at 14,000 rpm for 45 min. The supernatant was applied to a 5-ml Ni–nitrilotriacetic acid (NTA) column (Cytiva). The resin was washed with 10 column volumes lysis buffer and wash buffer (50 mM Tris pH 7.5, 50 mM NaCl, 20 mM imidazole, 10% glycerol, and freshly added 2 mM β-mercaptoethanol). The protein was eluted off the Ni-NTA column in elution buffer [50 mM tris (pH 7.5), 50 mM NaCl, 500 mM imidazole, and 10% glycerol] and brought onto a 5-ml HiTrap Q anion exchange column (Cytiva). The column was washed using anion A buffer [50 mM tris (pH 7.5), 50 mM NaCl, and 1 mM tris(2-carboxyethyl)phosphine hydrochloride (TCEP)]. Protein elution off the column was induced through a salt gradient between anion A buffer and anion B buffer [50 mM tris (pH 7.5), 1 M NaCl, and 1 mM TCEP]. The eluted protein was cleaved at the His6-TEV tag by the addition of His6-tagged TEV protease (0.10 mg/ml) at 4°C overnight. This cleavage cocktail was flowed through a 2-ml Ni-NTA gravity column (Thermo Fisher Scientific) to separate the cleaved protein from uncleaved protein and TEV protease. Last, the cleaved protein was purified by size exclusion chromatography (SEC) on a Superdex 200 10/300 gel filtration column (Cytiva) equilibrated with SEC buffer [20 mM Hepes (pH 7.5), 150 mM NaCl, and 10% glycerol]. Pure fractions were pooled and concentrated and flash-frozen in liquid N2 for long-term storage at −80°C.

Differential scanning fluorimetry

Purified protein stocks were thawed and diluted in DSF buffer [20 mM Hepes (pH 7.5), 50 mM NaCl, and 0.4% DMSO]. Nineteen microliters of buffer was added to a MicroAmp Fast Optical 96-well Reaction plate (Applied Biosystems, # 4346906). One microliters of 500× SYPRO Orange Protein Gel Stain (Thermo Fisher Scientific, catalog no. S-6650) was added to a final protein concentration of 10 μM and 25× SYPRO Orange. Melting curves were performed in an Applied Biosystems Step-One Plus RT-PCR thermocycler. Temperature measurements started at 15°C, and temperature was raised by 0.5°C every minute with continuous measurements of fluorescence (excitation: 472 nm; emission: 570 nm). Raw fluorescence values along with corresponding temperatures were analyzed using DSF-world and T_m values were calculated using delta relative fluorescence units (dRFU).

DiFMUP basal activity measurements

Initial rate measurements for the SHP2-catalyzed dephosphorylation of 6,8-difluoro-4-methylumbelliferyl phosphate (DiFMUP) were conducted at 37°C in DiFMUP buffer [60 mM Hepes (pH 7.2), 150 mM NaCl, 1 mM EDTA, and 0.05% Tween-20]. Reactions of 50 μl were set up in a black polystyrene flat bottom half area 96-well plate. A substrate concentration series of 31.25, 62.5, 125, 250, 500, 1000, 2000, and 4000 μM was used to determine catalytic constant (k_{cat}) and K_M (Michaelis constant). Reactions were started by the addition of appropriate amount of SHP2 WT and mutants (WT full-length: 2.5 nM; T507K full-length: 2.5 nM; Q510K full-length: 10 nM; Q510E: 2.5 nM; WT PTP: 1 nM; T507K PTP: 1 nM; Q510K PTP: 60 nM; Q510E PTP: 60 nM). Emitted fluorescence at 455 nm was recorded every 25 s in a span of 50 min using a BioTek Synergy Neo2 multimode reader.

The linear part of the reaction progress curve was determined by visual inspection and fit to a line. Slopes were converted from absorbance or fluorescence units as a function of time to product formation as a function of time using standard curves measured with the reaction products (6,8-difluoro-7-hydroxy-4-methylcoumarin). Last, these rates were corrected for enzyme concentration by dividing the values by the concentration of enzyme used in the experiment to yield V_0/[enzyme] in units of (s^{-1}). These corrected rates were plotted as a function of substrate concentration and fit to the Michaelis-Menten equation using nonlinear regression to determine catalytic parameters. Experiments were generally repeated at least three times, and the average and SD of all individual replicates are reported.

PTP activity assay against phosphopeptide substrates

Initial rate measurements of the dephosphorylation of phosphopeptide substrates were conducted at 37°C in freshly made reaction buffer [10 mM Hepes (pH 7.5), 150 mM NaCl, 1 mM TCEP, and 10% glycerol] using the EnzCheck Phosphate Assay Kit (Thermo Fisher Scientific) according to the manufacturer's instructions. Reactions of 50 μl were set up in a clear polystyrene flat bottom half area 96-well plate. A substrate concentration series of 0 to 1000 μM was used to determine k_{cat} and K_M. Reactions were started by the addition of 4000 nM SHP2. Absorbance at 360 nm was recorded every 8 s in a span of 6 min using a BioTek Synergy Neo2 multimode reader.

In all cases, the linear region of the reaction progress curve was determined by visual inspection and fit to a line. These slopes were converted from absorbance units as a function of time to product formation as a function of time using standard curves measured with inorganic phosphate. These rates were then corrected for enzyme concentration used in the experiment to yield V_0/[enzyme] in units of (s^{-1}). This was plotted as a function of substrate concentration and fit to the Michaelis-Menten equation using nonlinear regression to determine catalytic parameters. Experiments were repeated at least three times, and the average and SD of all individual replicates are reported.

Peptide synthesis and purification

The following peptides were synthesized with N-terminal acetyl and C-terminal carboxamide groups: (i) Sprouty1/pY53: Ac-GSNE-pY-TEGPS-NH2; (ii) Paxillin/pY118: Ac-EEHV-pY-SFPN-NH2; (iii) Gab1/pY589: Ac-DSEEN-pY-VPMNPNL-NH2; (iv) EGFR/pY992: Ac-DADE-pY-LIPQQG-NH2. All peptides were synthesized using 9-fluorenylmethoxycarbonyl (Fmoc) solid-phase peptide chemistry. All syntheses were carried out using the Liberty Blue automated microwave-assisted peptide synthesizer from CEM under nitrogen atmosphere, with standard manufacturer-recommended protocols. Peptides were synthesized on MBHA Rink amide resin (0.1 mmol scale). Each Nα-Fmoc–amino acid (6 equiv, 0.2 M) was activated with diisopropylcarbodiimide (1.0 M) and ethyl cyano(hydroxyamino)-acetate (OxymaPure, 1.0 M) in *N*,*N*-dimethylformamide (DMF) before coupling. Diisopropylethylamine (0.4 molar equiv, 0.4 M) was added to the OxymaPure solution following the CarboMAX method published by CEM. Each coupling cycle was done at 75°C for 15 s followed by 90°C for 110 s. Deprotection of the Fmoc group was performed in 20% (v/v) piperidine in DMF (75°C for 15 s then 90°C for 50 s). The resin was washed four times with DMF following Fmoc deprotection and after Nα-Fmoc amino acid coupling. All peptides were acetylated at the N terminus with 10% acetic anhydride/DMF and washed four times with DMF after the acetylation reaction.

Following peptide synthesis, the resin was washed three times with DMF, dichloromethane, and methanol (MeOH) and dried under reduced pressure overnight. The peptides were cleaved from resin, and the side chains were simultaneously deprotected in 95% (v/v) trifluoroacetic acid (TFA), 2.5% (v/v) water, and 2.5% (v/v) triisopropylsilane, in a ratio of 10 μl of cleavage cocktail per milligram of resin. The cleavage-resin mixture was incubated at room temperature for 90 min, with agitation. The cleaved peptides were precipitated in cold diethyl ether, pelleted, and dried under air. The peptides were dissolved in 50% (v/v) acetonitrile/water solution and filtered from resin. The filtrate was freeze-dried for downstream purification.

The crude peptide mixture was purified using reverse-phase high-performance liquid chromatography on a preparatory C18 column (Waters, XBridge Peptide BEH C18 OBD Prep Column, 19 mm by 150 mm, 5 μm). Flow rate was maintained at 17 ml/min with solvents A [water, 0.1% (v/v) TFA] and B [acetonitrile, 0.1% (v/v) TFA]. Peptides were generally purified over a 23-min linear gradient from 0 to 70% solvent B. Peptide purity was assessed using an analytical column (Agilent, ZORBAX 300 SB-C18, 4.6 mm by 150 mm, 5 μm) at a flow rate of 1 ml/min over a 0 to 90% solvent B gradient in 30 min. All peptides were determined to be ≥95% pure by peak integration. The identities of the peptides were confirmed using mass spectrometry (Waters Xevo G2-XS QTOF). Pure peptides were lyophilized and redissolved in tris buffer (100 mM, pH 8.0) as needed for experiments.

Supplementary Materials

This PDF file includes:
Figs. S1 to S6
Legends for tables S1 to S3

Other Supplementary Material for this manuscript includes the following:
Tables S1 to S3

REFERENCES

1. E. H. Estey, Acute myeloid leukemia: 2019 update on risk-stratification and management. *Am. J. Hematol.* **93**, 1267–1291 (2018).
2. S. Fobare, J. Kohlschmidt, H. G. Ozer, K. Mrózek, D. Nicolet, A. S. Mims, R. Garzon, J. S. Blachly, S. Orwick, A. J. Carroll, R. M. Stone, E. S. Wang, J. E. Kolitz, B. L. Powell, C. C. Oakes, A.-K. Eisfeld, E. Hertlein, J. C. Byrd, Molecular, clinical, and prognostic implications of PTPN11 mutations in acute myeloid leukemia. *Blood Adv.* **6**, 1371–1380 (2022).
3. A. K. Gupta, J. P. Meena, A. Chopra, P. Tanwar, R. Seth, Juvenile myelomonocytic leukemia-A comprehensive review and recent advances in management. *Am. J. Blood Res.* **11**, 1–21 (2021).
4. M. Bentires-Alj, J. G. Paez, F. S. David, H. Keilhack, B. Halmos, K. Naoki, J. M. Maris, A. Richardson, A. Bardelli, D. J. Sugarbaker, W. G. Richards, J. Du, L. Girard, J. D. Minna, M. L. Loh, D. E. Fisher, V. E. Velculescu, B. Vogelstein, M. Meyerson, W. R. Sellers, B. G. Neel, Activating mutations of the noonan syndrome-associated ***SHP2/PTPN11*** gene in human solid tumors and adult acute myelogenous leukemia. *Cancer Res.* **64**, 8816–8820 (2004).
5. D. Miyamoto, M. Miyamoto, A. Takahashi, Y. Yomogita, H. Higashi, S. Kondo, M. Hatakeyama, Isolation of a distinct class of gain-of-function SHP-2 mutants with oncogenic RAS-like transforming activity from solid tumors. *Oncogene* **27**, 3508–3515 (2008).
6. C. W. Brennan, R. G. W. Verhaak, A. McKenna, B. Campos, H. Noushmehr, S. R. Salama, S. Zheng, D. Chakravarty, J. Z. Sanborn, S. H. Berman, R. Beroukhim, B. Bernard, C.-J. Wu, G. Genovese, I. Shmulevich, J. Barnholtz-Sloan, L. Zou, R. Vegesna, S. A. Shukla, G. Ciriello, W. K. Yung, W. Zhang, C. Sougnez, T. Mikkelsen, K. Aldape, D. D. Bigner, E. G. Van Meir, M. Prados, A. Sloan, K. L. Black, J. Eschbacher, G. Finocchiaro, W. Friedman, D. W. Andrews, A. Guha, M. Iacocca, B. P. O'Neill, G. Foltz, J. Myers, D. J. Weisenberger, R. Penny, R. Kucherlapati, C. M. Perou, D. N. Hayes, R. Gibbs, M. Marra, G. B. Mills, E. Lander, P. Spellman, R. Wilson, C. Sander, J. Weinstein, M. Meyerson, S. Gabriel, P. W. Laird, D. Haussler, G. Getz, L. Chin, TCGA Research Network, The somatic genomic landscape of glioblastoma. *Cell* **155**, 462–477 (2013).
7. M. Tartaglia, E. L. Mehler, R. Goldberg, G. Zampino, H. G. Brunner, H. Kremer, I. van der Burgt, A. H. Crosby, A. Ion, S. Jeffery, K. Kalidas, M. A. Patton, R. S. Kucherlapati, B. D. Gelb, Mutations in PTPN11, encoding the protein tyrosine phosphatase SHP-2, cause Noonan syndrome. *Nat. Genet.* **29**, 465–468 (2001).
8. A. E. Roberts, T. Araki, K. D. Swanson, K. T. Montgomery, T. A. Schiripo, V. A. Joshi, L. Li, Y. Yassin, A. M. Tamburino, B. G. Neel, R. S. Kucherlapati, Germline gain-of-function mutations in SOS1 cause Noonan syndrome. *Nat. Genet.* **39**, 70–74 (2007).
9. S. Schubbert, M. Zenker, S. L. Rowe, S. Böll, C. Klein, G. Bollag, I. van der Burgt, L. Musante, V. Kalscheuer, L.-E. Wehner, H. Nguyen, B. West, K. Y. J. Zhang, E. Sistermans, A. Rauch, C. M. Niemeyer, K. Shannon, C. P. Kratz, Germline KRAS mutations cause Noonan syndrome. *Nat. Genet.* **38**, 331–336 (2006).
10. B. Pandit, A. Sarkozy, L. A. Pennacchio, C. Carta, K. Oishi, S. Martinelli, E. A. Pogna, W. Schackwitz, A. Ustaszewska, A. Landstrom, J. M. Bos, S. R. Ommen, G. Esposito, F. Lepri, C. Faul, P. Mundel, J. P. López Siguero, R. Tenconi, A. Selicorni, C. Rossi, L. Mazzanti, I. Torrente, B. Marino, M. C. Digilio, G. Zampino, M. J. Ackerman, B. Dallapiccola, M. Tartaglia, B. D. Gelb, Gain-of-function RAF1 mutations cause Noonan and LEOPARD syndromes with hypertrophic cardiomyopathy. *Nat. Genet.* **39**, 1007–1012 (2007).
11. M. A. Razzaque, T. Nishizawa, Y. Komoike, H. Yagi, M. Furutani, R. Amo, M. Kamisago, K. Momma, H. Katayama, M. Nakagawa, Y. Fujiwara, M. Matsushima, K. Mizuno, M. Tokuyama, H. Hirota, J. Muneuchi, T. Higashinakagawa, R. Matsuoka, Germline gain-of-function mutations in RAF1 cause Noonan syndrome. *Nat. Genet.* **39**, 1013–1017 (2007).
12. B. D. Gelb, M. Tartaglia, "Noonan syndrome with multiple lentigines" in *GeneReviews®*, M. P. Adam, J. Feldman, G. M. Mirzaa, R. A. Pagon, S. E. Wallace, A. Amemiya, Eds. (Univ. of Washington, 1993; http://ncbi.nlm.nih.gov/books/NBK1383/).
13. M. C. Digilio, E. Conti, A. Sarkozy, R. Mingarelli, T. Dottorini, B. Marino, A. Pizzuti, B. Dallapiccola, Grouping of multiple-lentigines/LEOPARD and Noonan syndromes on the PTPN11 gene. *Am. J. Hum. Genet.* **71**, 389–394 (2002).
14. E. Legius, C. Schrander-Stumpel, E. Schollen, C. Pulles-Heintzberger, M. Gewillig, J.-P. Fryns, PTPN11 mutations in LEOPARD syndrome. *J. Med. Genet.* **39**, 571–574 (2002).
15. Z. Guo, Y. Duan, K. Sun, T. Zheng, J. Liu, S. Xu, J. Xu, Advances in SHP2 tunnel allosteric inhibitors and bifunctional molecules. *Eur. J. Med. Chem.* **275**, 116579 (2024).
16. X. Yuan, H. Bu, J. Zhou, C.-Y. Yang, H. Zhang, Recent advances of SHP2 inhibitors in cancer therapy: Current development and clinical application. *J. Med. Chem.* **63**, 11368–11396 (2020).
17. M. Dance, A. Montagner, J.-P. Salles, A. Yart, P. Raynal, The molecular functions of Shp2 in the Ras/Mitogen-activated protein kinase (ERK1/2) pathway. *Cell. Signal.* **20**, 453–459 (2008).

18. A. Montagner, A. Yart, M. Dance, B. Perret, J.-P. Salles, P. Raynal, A novel role for Gab1 and SHP2 in epidermal growth factor-induced Ras activation. *J. Biol. Chem.* **280**, 5350–5360 (2005).
19. S. Bunda, K. Burrell, P. Heir, L. Zeng, A. Alamsahebpour, Y. Kano, B. Raught, Z.-Y. Zhang, G. Zadeh, M. Ohh, Inhibition of SHP2-mediated dephosphorylation of Ras suppresses oncogenesis. *Nat. Commun.* **6**, 8859 (2015).
20. G. A. Rodrigues, M. Falasca, Z. Zhang, S. H. Ong, J. Schlessinger, A novel positive feedback loop mediated by the docking protein Gab1 and phosphatidylinositol 3-kinase in epidermal growth factor receptor signaling. *Mol. Cell. Biol.* **20**, 1448–1459 (2000).
21. Y. M. Agazie, M. J. Hayman, Molecular mechanism for a role of SHP2 in epidermal growth factor receptor signaling. *Mol. Cell. Biol.* **23**, 7875–7886 (2003).
22. Y. M. Agazie, N. Movilla, I. Ischenko, M. J. Hayman, The phosphotyrosine phosphatase SHP2 is a critical mediator of transformation induced by the oncogenic fibroblast growth factor receptor 3. *Oncogene* **22**, 6909–6918 (2003).
23. T. S. Batth, M. Papetti, A. Pfeiffer, M. A. X. Tollenaere, C. Francavilla, J. V. Olsen, Large-scale phosphoproteomics reveals Shp-2 phosphatase-dependent regulators of Pdgf receptor signaling. *Cell Rep.* **22**, 2784–2796 (2018).
24. R. J. Salmond, D. R. Alexander, SHP2 forecast for the immune system: Fog gradually clearing. *Trends Immunol.* **27**, 154–160 (2006).
25. Z. Jiang, A. E. Van Vlimmeren, D. Karandur, A. Semmelman, N. H. Shah, Deep mutational scanning of a multi-domain signaling protein reveals mechanisms of regulation and pathogenicity. biorxiv [Preprint] (2024). https://doi.org/10.1101/2024.05.13.593907.
26. S. Martinelli, P. Torreri, M. Tinti, L. Stella, G. Bocchinfuso, E. Flex, A. Grottesi, M. Ceccarini, A. Palleschi, G. Cesareni, L. Castagnoli, T. C. Petrucci, B. D. Gelb, M. Tartaglia, Diverse driving forces underlie the invariant occurrence of the T42A, E139D, I282V and T468M SHP2 amino acid substitutions causing Noonan and LEOPARD syndromes. *Hum. Mol. Genet.* **17**, 2018–2029 (2008).
27. A. E. van Vlimmeren, R. Voleti, C. A. Chartier, Z. Jiang, D. Karandur, P. A. Humphries, W.-L. Lo, N. H. Shah, The pathogenic T42A mutation in SHP2 rewires the interaction specificity of its N-terminal regulatory domain. *Proc. Natl. Acad. Sci. U.S.A.* **121**, e2407159121 (2024).
28. H. Keilhack, F. S. David, M. McGregor, L. C. Cantley, B. G. Neel, Diverse biochemical properties of Shp2 mutants. *J. Biol. Chem.* **280**, 30984–30993 (2005).
29. Z.-H. Yu, R.-Y. Zhang, C. D. Walls, L. Chen, S. Zhang, L. Wu, S. Liu, Z.-Y. Zhang, Molecular basis of gain-of-function LEOPARD syndrome-associated SHP2 mutations. *Biochemistry* **53**, 4136–4151 (2014).
30. M. Marasco, A. Berteotti, J. Weyershaeuser, N. Thorausch, J. Sikorska, J. Krausze, H. J. Brandt, J. Kirkpatrick, P. Rios, W. W. Schamel, M. Köhn, T. Carlomagno, Molecular mechanism of SHP2 activation by PD-1 stimulation. *Sci. Adv.* **6**, eaay4458 (2020).
31. J. R. LaRochelle, M. Fodor, V. Vemulapalli, M. Mohseni, P. Wang, T. Stams, M. J. LaMarche, R. Chopra, M. G. Acker, S. C. Blacklow, Structural reorganization of SHP2 by oncogenic mutations and implications for oncoprotein resistance to allosteric inhibition. *Nat. Commun.* **9**, 4508 (2018).
32. Z.-H. Yu, J. Xu, C. D. Walls, L. Chen, S. Zhang, R. Zhang, L. Wu, L. Wang, S. Liu, Z.-Y. Zhang, Structural and mechanistic insights into LEOPARD syndrome-associated SHP2 mutations. *J. Biol. Chem.* **288**, 10472–10482 (2013).
33. J.-S. Yi, S. Perla, L. Enyenihi, A. M. Bennett, Tyrosyl phosphorylation of PZR promotes hypertrophic cardiomyopathy in PTPN11-associated Noonan syndrome with multiple lentigines. *JCI Insight* **5**, e137753, 137753 (2020).
34. A. E. Van Vlimmeren, L. C. Tang, Z. Jiang, A. Iyer, R. Voleti, K. Krismer, J. T. Gaublomme, M. Jovanovic, N. H. Shah, Proximity-labeling proteomics reveals remodeled interactomes and altered localization of pathogenic SHP2 variants. *EMBO Rep.* **27**, 793–826 (2026).
35. Y. Zhao, Z. Chang, B. Hu, Q. Zhang, D. Zhang, C. He, Y. Guo, Z. Peng, C. Chen, Y. Chen, Transcriptome analysis reveals effects of leukemogenic SHP2 mutations in biosynthesis of amino acids signaling. *Front. Oncol.* **13**, 1090542 (2023).
36. H. S. Kim, S. M. Grimes, T. Chen, A. Sathe, B. T. Lau, G.-H. Hwang, S. Bae, H. P. Ji, Direct measurement of engineered cancer mutations and their transcriptional phenotypes in single cells. *Nat. Biotechnol.* **42**, 1254–1262 (2024).
37. O. Ursu, J. T. Neal, E. Shea, P. I. Thakore, L. Jerby-Arnon, L. Nguyen, D. Dionne, C. Diaz, J. Bauman, M. M. Mosaad, C. Fagre, A. Lo, M. McSharry, A. O. Giacomelli, S. H. Ly, O. Rozenblatt-Rosen, W. C. Hahn, A. J. Aguirre, A. H. Berger, A. Regev, J. S. Boehm, Massively parallel phenotyping of coding variants in cancer with Perturb-seq. *Nat. Biotechnol.* **40**, 896–905 (2022).
38. Y. Baglaenko, Z. Mu, M. Curtis, H. M. Mire, V. Jayanthi, M. Al Suqri, C. Liu, R. Agnew, A. Nathan, A. Y. Mah-Som, D. R. Liu, G. A. Newby, S. Raychaudhuri, Precisely defining disease variant effects in CRISPR-edited single cells. *Nature* **646**, 117–125 (2025).
39. K. Ozturk, R. Panwala, J. Sheen, K. Ford, N. Jayne, A. Portell, D.-E. Zhang, S. Hutter, T. Haferlach, T. Ideker, P. Mali, H. Carter, Interface-guided phenotyping of coding variants in the transcription factor RUNX1. *Cell Rep.* **43**, 114436 (2024).
40. J. D. Martin-Rufino, N. Castano, M. Pang, E. I. Grody, S. Joubran, A. Caulier, L. Wahlster, T. Li, X. Qiu, A. M. Riera-Escandell, G. A. Newby, A. Al'Khafaji, S. Chaudhary, S. Black, C. Weng, G. Munson, D. R. Liu, M. W. Wlodarski, K. Sims, J. H. Oakley, R. M. Fasano, R. J. Xavier, E. S. Lander, D. E. Klein, V. G. Sankaran, Massively parallel base editing to map variant effects in human hematopoiesis. *Cell* **186**, 2456–2474.e24 (2023).
41. S. R. Srivatsan, J. L. McFaline-Figueroa, V. Ramani, L. Saunders, J. Cao, J. Packer, H. A. Pliner, D. L. Jackson, R. M. Daza, L. Christiansen, F. Zhang, F. Steemers, J. Shendure, C. Trapnell, Massively multiplex chemical transcriptomics at single-cell resolution. *Science* **367**, 45–51 (2020).
42. R. M. Giglio, N. Hou, A. Wyatt, J. Hong, L. Shi, M. Vaikunthan, H. Fuchs, J. P. Nima, S. W. Malinowski, K. L. Ligon, J. R. McFaline-Figueroa, N. Yosef, E. Azizi, J. L. McFaline-Figueroa, A heterogeneous pharmaco-transcriptomic landscape induced by targeting a single oncogenic kinase. biorxiv [Preprint] (2024). https://doi.org/10.1101/2024.04.08.587960.
43. D. P. Cook, B. C. Vanderhyden, Context specificity of the EMT transcriptional response. *Nat. Commun.* **11**, 2142 (2020).
44. B. K. Martin, C. Qiu, E. Nichols, M. Phung, R. Green-Gladden, S. Srivatsan, R. Blecher-Gonen, B. J. Beliveau, C. Trapnell, J. Cao, J. Shendure, Optimized single-nucleus transcriptional profiling by combinatorial indexing. *Nat. Protoc.* **18**, 188–207 (2023).
45. J. Cao, M. Spielmann, X. Qiu, X. Huang, D. M. Ibrahim, A. J. Hill, F. Zhang, S. Mundlos, L. Christiansen, F. J. Steemers, C. Trapnell, J. Shendure, The single-cell transcriptional landscape of mammalian organogenesis. *Nature* **566**, 496–502 (2019).
46. A. Sziraki, Z. Lu, J. Lee, G. Banyai, S. Anderson, A. Abdulraouf, E. Metzner, A. Liao, J. Banfelder, A. Epstein, C. Schaefer, Z. Xu, Z. Zhang, L. Gan, P. T. Nelson, W. Zhou, J. Cao, A global view of aging and Alzheimer's pathogenesis-associated cell population dynamics and molecular signatures in human and mouse brains. *Nat. Genet.* **55**, 2104–2116 (2023).
47. J. L. McFaline-Figueroa, S. Srivatsan, A. J. Hill, M. Gasperini, D. L. Jackson, L. Saunders, S. Domcke, S. G. Regalado, P. Lazarchuck, S. Alvarez, R. J. Monnat Jr., J. Shendure, C. Trapnell, Multiplex single-cell chemical genomics reveals the kinase dependence of the response to targeted therapy. *Cell Genom.* **4**, 100487 (2024).
48. J. W. Tullai, M. E. Schaffer, S. Mullenbrock, G. Sholder, S. Kasif, G. M. Cooper, Immediate-early and delayed primary response genes are distinct in function and genomic architecture. *J. Biol. Chem.* **282**, 23981–23995 (2007).
49. I. Amit, A. Citri, T. Shay, Y. Lu, M. Katz, F. Zhang, G. Tarcic, D. Siwak, J. Lahad, J. Jacob-Hirsch, N. Amariglio, N. Vaisman, E. Segal, G. Rechavi, U. Alon, G. B. Mills, E. Domany, Y. Yarden, A module of negative feedback regulators defines growth factor signaling. *Nat. Genet.* **39**, 503–512 (2007).
50. Y.-N. P. Chen, M. J. LaMarche, H. M. Chan, P. Fekkes, J. Garcia-Fortanet, M. G. Acker, B. Antonakos, C. H.-T. Chen, Z. Chen, V. G. Cooke, J. R. Dobson, Z. Deng, F. Fei, B. Firestone, M. Fodor, C. Fridrich, H. Gao, D. Grunenfelder, H.-X. Hao, J. Jacob, S. Ho, K. Hsiao, Z. B. Kang, R. Karki, M. Kato, J. Larrow, L. R. La Bonte, F. Lenoir, G. Liu, S. Liu, D. Majumdar, M. J. Meyer, M. Palermo, L. Perez, M. Pu, E. Price, C. Quinn, S. Shakya, M. D. Shultz, J. Slisz, K. Venkatesan, P. Wang, M. Warmuth, S. Williams, G. Yang, J. Yuan, J.-H. Zhang, P. Zhu, T. Ramsey, N. J. Keen, W. R. Sellers, T. Stams, P. D. Fortin, Allosteric inhibition of SHP2 phosphatase inhibits cancers driven by receptor tyrosine kinases. *Nature* **535**, 148–152 (2016).
51. W. Yang, J. Wang, D. C. Moore, H. Liang, M. Dooner, Q. Wu, R. Terek, Q. Chen, M. G. Ehrlich, P. J. Quesenberry, B. G. Neel, Ptpn11 deletion in a novel progenitor causes metachondromatosis by inducing hedgehog signalling. *Nature* **499**, 491–495 (2013).
52. A. Papadopoulou, E. Bountouvi, Skeletal defects and bone metabolism in Noonan, Costello and cardio-facio-cutaneous syndromes. *Front. Endocrinol. (Lausanne)* **14**, 1231828 (2023).
53. L. A. Ehrman, D. Nardini, S. Ehrman, T. A. Rizvi, J. Gulick, M. Krenz, B. Dasgupta, J. Robbins, N. Ratner, M. Nakafuku, R. R. Waclaw, The protein tyrosine phosphatase Shp2 is required for the generation of oligodendrocyte progenitor cells and myelination in the mouse telencephalon. *J. Neurosci.* **34**, 3767–3778 (2014).
54. A. Liberzon, C. Birger, H. Thorvaldsdóttir, M. Ghandi, J. P. Mesirov, P. Tamayo, The Molecular Signatures Database (MSigDB) hallmark gene set collection. *Cell Syst.* **1**, 417–425 (2015).
55. A. Toto, F. Malagrinò, L. Visconti, F. Troilo, S. Gianni, Unveiling the molecular basis of the noonan syndrome-causing mutation T42A of SHP2. *Int. J. Mol. Sci.* **21**, 461 (2020).
56. C. P. Kratz, The mutational spectrum of PTPN11 in juvenile myelomonocytic leukemia and Noonan syndrome/myeloproliferative disease. *Blood* **106**, 2183–2185 (2005).
57. A. Sarkozy, E. Conti, M. C. Digilio, B. Marino, E. Morini, G. Pacileo, M. Wilson, R. Calabrò, A. Pizzuti, B. Dallapiccola, Clinical and molecular analysis of 30 patients with multiple lentigines LEOPARD syndrome. *J. Med. Genet.* **41**, e68 (2004).
58. R.-Y. Zhang, Z.-H. Yu, L. Chen, C. D. Walls, S. Zhang, L. Wu, Z.-Y. Zhang, Mechanistic insights explain the transforming potential of the T507K substitution in the protein-tyrosine phosphatase SHP2. *J. Biol. Chem.* **295**, 6187–6201 (2020).
59. L. McInnes, J. Healy, J. Melville, UMAP: Uniform Manifold Approximation and Projection for Dimension Reduction. arXiv [stat.ML] (2018). https://doi.org/10.48550/ARXIV.1802.03426.
60. P. Boyeau, J. Hong, A. Gayoso, M. Kim, J. L. McFaline-Figueroa, M. I. Jordan, E. Azizi, C. Ergen, N. Yosef, Deep generative modeling of sample-level heterogeneity in single-cell genomics. *Nat. Methods* **22**, 2264–2274 (2025).
61. V. A. Traag, L. Waltman, N. J. van Eck, From Louvain to Leiden: Guaranteeing well-connected communities. *Sci. Rep.* **9**, 5233 (2019).
62. D. Zhang, R. Zhu, H. Zhang, C.-H. Zheng, J. Xia, MGDB: A comprehensive database of genes involved in melanoma. *Database (Oxford)* **2015**, bav097 (2015).
63. B. Pan, X. Lin, L. Zhang, W. Hong, Y. Zhang, Long noncoding RNA X-inactive specific transcript promotes malignant melanoma progression and oxaliplatin resistance. *Melanoma Res.* **29**, 254–262 (2019).

64. X. Rui, Y. Xu, X. Jiang, W. Ye, Y. Huang, J. Jiang, Long non-coding RNA C5orf66-AS1 promotes cell proliferation in cervical cancer by targeting miR-637/RING1 axis. *Cell Death Dis.* **9**, 1175 (2018).
65. S. Zhu, J. Sun, X. Liu, H. Shao, C. Feng, Z. Wang, X. Zheng, S. Wei, CTCF-induced lncRNA C5orf66-AS1 facilitates the progression of triple-negative breast cancer via Sponging miR-149-5p to up-regulate CTCF and CTNNB1 to activate Wnt/β-catenin pathway. *Mol. Cell. Biol.* **42**, e00188-21 (2022).
66. T. Lu, H. Liu, G. You, Long non-coding RNA C5orf66-AS1 prevents oral squamous cell carcinoma through inhibiting cell growth and metastasis. *Int. J. Mol. Med.* **42**, 3291–3299 (2018).
67. G. Wei, H. Luo, Y. Sun, J. Li, L. Tian, W. Liu, L. Liu, J. Luo, J. He, R. Chen, Transcriptome profiling of esophageal squamous cell carcinoma reveals a long noncoding RNA acting as a tumor suppressor. *Oncotarget* **6**, 17065–17080 (2015).
68. M. Sang, L. Wang, C. Ding, X. Zhou, B. Wang, L. Wang, Y. Lian, B. Shan, Melanoma-associated antigen genes - an update. *Cancer Lett.* **302**, 85–90 (2011).
69. W.-G. Shen, Q.-Y. Xue, Y.-D. Wu, B.-S. Hu, J. Zhu, Y. Zhang, Q. Su, Melanoma-associated antigen family protein-D1 regulation of tumor cell migration, adhesion to endothelium, and actin structures reorganization in response to hypoxic stress. *Cell Commun. Adhes.* **14**, 21–31 (2007).
70. S. Hussein, A. S. Abdelazem, S. Abdelmoneem, A.-S. M. Abdelnabi, T. Khamis, A. A. Obaya, H. F. Ebian, Evaluation of miRNA 223/125a and COBLL1 expressions and ROR-1 levels as reliable markers in B- chronic lymphocytic leukemia. *Asian Pac. J. Cancer Prev.* **23**, 2735–2742 (2022).
71. K.-I. Takayama, T. Suzuki, T. Fujimura, S. Takahashi, S. Inoue, COBLL1 modulates cell morphology and facilitates androgen receptor genomic binding in advanced prostate cancer. *Proc. Natl. Acad. Sci. U.S.A.* **115**, 4975–4980 (2018).
72. M. Tartaglia, S. Martinelli, L. Stella, G. Bocchinfuso, E. Flex, V. Cordeddu, G. Zampino, I. Van Der Burgt, A. Palleschi, T. C. Petrucci, M. Sorcini, C. Schoch, R. Foà, P. D. Emanuel, B. D. Gelb, Diversity and functional consequences of germline and somatic PTPN11 mutations in human disease. *Am. J. Hum. Genet.* **78**, 279–290 (2006).
73. J. Abramson, J. Adler, J. Dunger, R. Evans, T. Green, A. Pritzel, O. Ronneberger, L. Willmore, A. J. Ballard, J. Bambrick, S. W. Bodenstein, D. A. Evans, C.-C. Hung, M. O'Neill, D. Reiman, K. Tunyasuvunakool, Z. Wu, A. Žemgulytė, E. Arvaniti, C. Beattie, O. Bertolli, A. Bridgland, A. Cherepanov, M. Congreve, A. I. Cowen-Rivers, A. Cowie, M. Figurnov, F. B. Fuchs, H. Gladman, R. Jain, Y. A. Khan, C. M. R. Low, K. Perlin, A. Potapenko, P. Savy, S. Singh, A. Stecula, A. Thillaisundaram, C. Tong, S. Yakneen, E. D. Zhong, M. Zielinski, A. Žídek, V. Bapst, P. Kohli, M. Jaderberg, D. Hassabis, J. M. Jumper, Accurate structure prediction of biomolecular interactions with AlphaFold 3. *Nature* **630**, 493–500 (2024).
74. A. Serbina, A. C. Bishop, Quantitation of autoinhibitory defects in pathogenic SHP2 mutants by differential scanning fluorimetry. *Anal. Biochem.* **680**, 115300 (2023).
75. Z. J. Hauseman, F. Stauffer, K. S. Beyer, S. Mollé, E. Cavicchioli, J.-R. Marchand, M. Fodor, J. Viscomi, A. Dhembi, S. Katz, B. Faggion, M. Lanter, G. Kerr, D. Schildknecht, C. Handl, D. Maddalo, C. Pissot Soldermann, J. Brady, O. Shrestha, Z. Nguyen, L. Leder, G. Cremosnik, S. Lopez Romero, U. Hassiepen, T. Stams, M. Linder, G. G. Galli, D. A. Guthy, D. A. King, S.-M. Maira, C. R. Thoma, V. Ehmke, L. Tordella, Targeting the SHOC2-RAS interaction in RAS-mutant cancers. *Nature* **642**, 232–241 (2025).
76. J. C. Hunter, A. Manandhar, M. A. Carrasco, D. Gurbani, S. Gondi, K. D. Westover, Biochemical and structural analysis of common cancer-associated KRAS mutations. *Mol. Cancer Res.* **13**, 1325–1335 (2015).
77. A. Subramanian, P. Tamayo, V. K. Mootha, S. Mukherjee, B. L. Ebert, M. A. Gillette, A. Paulovich, S. L. Pomeroy, T. R. Golub, E. S. Lander, J. P. Mesirov, Gene set enrichment analysis: A knowledge-based approach for interpreting genome-wide expression profiles. *Proc. Natl. Acad. Sci. U.S.A.* **102**, 15545–15550 (2005).
78. T. A. Ahmed, C. Adamopoulos, Z. Karoulia, X. Wu, R. Sachidanandam, S. A. Aaronson, P. I. Poulikakos, SHP2 drives adaptive resistance to ERK signaling inhibition in molecularly defined subsets of ERK-dependent tumors. *Cell Rep.* **26**, 65–78.e5 (2019).
79. H. Lu, C. Liu, H. Huynh, T. B. U. Le, M. J. LaMarche, M. Mohseni, J. A. Engelman, P. S. Hammerman, G. Caponigro, H.-X. Hao, Resistance to allosteric SHP2 inhibition in FGFR-driven cancers through rapid feedback activation of FGFR. *Oncotarget* **11**, 265–281 (2020).
80. A. O. Walter, Z.-Y. Peng, C. A. Cartwright, The Shp-2 tyrosine phosphatase activates the Src tyrosine kinase by a non-enzymatic mechanism. *Oncogene* **18**, 1911–1920 (1999).
81. J. Paardekooper Overman, J.-S. Yi, M. Bonetti, M. Soulsby, C. Preisinger, M. P. Stokes, L. Hui, J. C. Silva, J. Overvoorde, P. Giansanti, A. J. R. Heck, M. I. Kontaridis, J. Den Hertog, A. M. Bennett, PZR coordinates Shp2 Noonan and LEOPARD syndrome signaling in zebrafish and mice. *Mol. Cell. Biol.* **34**, 2874–2889 (2014).
82. S. Perla, A. L. Stiegler, J.-S. Yi, L. Enyenihi, L. Zhang, M. Riaz, E. An, Y. Qyang, T. J. Boggon, A. M. Bennett, SHP2 genetic variants in NSML-associated RASopathies disrupt the PZR-IRX transcription factor signaling axis. *Proc. Natl. Acad. Sci. U.S.A.* **122**, e2503631122 (2025).
83. D. M. Fowler, J. J. Stephany, S. Fields, Measuring the activity of protein variants on a large scale using deep mutational scanning. *Nat. Protoc.* **9**, 2267–2284 (2014).

Acknowledgments: We would like to thank the members of the Shah and McFaline-Figueroa laboratories for scientific insights and helpful discussions, in particular, N. Hou for MrVI guidance. **Funding:** This research was funded by NIH/NIGMS grant R35GM138014 to N.H.S. J.L.M.-F. acknowledges support from grants from the NIH (R35HG011941) and the NSF (2146007). These studies used the resources of the Cancer Center Sequencing Core Facility at Columbia University funded in part through Center Grant P30CA013696. **Author contributions:** Conceptualization: A.E.V., R.M.G., J.L.M.-F., and N.H.S. Methodology: A.E.V., R.M.G., J.L.M.-F., and N.H.S. Investigation: A.E.V., R.M.G., Z.J., and M.L. Validation: A.E.V., Z.J., J.L.M.-F., and N.H.S. Formal analysis: A.E.V., R.M.G., Z.J., and N.H.S. Data curation: A.E.V. and R.M.G. Visualization: A.E.V., R.M.G., J.L.M.-F., and N.H.S. Project administration: A.E.V., J.L.M.-F., and N.H.S. Resources: J.L.M.-F., and N.H.S. Funding acquisition: J.L.M.-F. and N.H.S. Supervision: J.L.M.-F. and N.H.S. Writing—original draft: A.E.V., R.M.G., J.L.M.-F., and N.H.S. Writing—review and editing: A.E.V., R.M.G., Z.J., M.L., J.L.M.-F., and N.H.S. **Competing interests:** The authors declare that they have no competing interests. **Data, code, and materials availability:** All data and code needed to evaluate and reproduce the results in the paper are present in the paper and/or the Supplementary Materials. Raw and processed sequencing data can be accessed and downloaded from NCBI GEO under accession number GSE300865 (https://ncbi.nlm.nih.gov/geo/query/acc.cgi?acc=GSE300865). The code necessary to reproduce the analyses in this study can be found at https://doi.org/10.5281/zenodo.18825549. Plasmids for the mammalian and bacterial expression of SHP2 variants were generated for this study. These plasmids can be found on Addgene (https://addgene.org/browse/article/28261464/).

Submitted 25 July 2025
Accepted 14 April 2026
Published 22 May 2026
10.1126/sciadv.aea9389
